# hnRNP Q and hnRNP A1 Regulate the Translation of Cofilin in Response to Transient Oxygen–Glucose Deprivation in Hippocampal Neurons

**DOI:** 10.3390/cells10123567

**Published:** 2021-12-17

**Authors:** Sung Wook Kim, In Kyung Hong, Mingee Kim, Yun Seon Song, Kyong-Tai Kim

**Affiliations:** 1 Department of Life Sciences, Pohang University of Science and Technology (POSTECH), Pohang 37673, Korea; kimsw@postech.ac.kr; 2 College of Pharmacy, Sookmyung Women’s University, Seoul 04310, Korea; on579@hanmail.net (I.K.H.); gee5424@sookmyung.ac.kr (M.K.)

**Keywords:** oxygen–glucose deprivation, cofilin–actin aggregates, mRNA translation, neurodegeneration, RNA-binding proteins

## Abstract

Protein aggregates of cofilin and actin have been found in neurons under oxygen–glucose deprivation. However, the regulatory mechanism behind the expression of *Cfl1* during oxygen–glucose deprivation remains unclear. Here, we found that heterogeneous nuclear ribonucleoproteins (hnRNP) Q and hnRNP A1 regulate the translation of *Cfl1* mRNA, and formation of cofilin–actin aggregates. The interaction between hnRNP A1 and *Cfl1* mRNA was interrupted by hnRNP Q under normal conditions, while the changes in the expression and localization of hnRNP Q and hnRNP A1 increased such interaction, as did the translation of *Cfl1* mRNA under oxygen–glucose deprived conditions. These findings reveal a new translational regulatory mechanism of *Cfl1* mRNA in hippocampal neurons under oxygen–glucose deprivation.

## 1. Introduction

A loss of neurons in our brain could lead to severe defects in our bodily functions [1]. Several factors, such as protein aggregates and inflammation, could lead to the degeneration of neurons [2,3]. Additionally, since our brain is an organ that uses a great deal of energy, deprivation of oxygen and glucose could also cause severe neurodegeneration [4,5]. Within a few minutes of oxygen–glucose deprivation (OGD), the electrochemical gradient in neurons collapses, leading to their degeneration [6].

One of the hallmarks found in neurodegeneration caused by oxygen–glucose deprivation is cofilin–actin aggregates [7]. There are several types of cofilin–actin aggregates found in neurodegenerative disease patients [8]. These cofilin–actin aggregates are toxic to neurons because they interfere with the intracellular trafficking system, which induces synaptic loss [9]. Cofilin–actin aggregates are known to be formed when the intracellular level of reactive oxygen species (ROS) rises due to oxidative stress [10,11]. Interestingly, a previous study also showed that upregulated expression of *Cofilin* (*Cfl1*) could induce the formation of the aggregates [12]. Despite its importance, studies on the regulatory mechanism behind the expression of *Cfl1* under both normal and disease condition are rare. We have shown that the local translation of *Cfl1* mRNA preferentially happens through an internal ribosome entry site (IRES) [13]. However, the relationship between previously found mechanisms and brain disease remains unclear.

To address such a challenge, we used primary hippocampal neurons cultured in chemically oxygen–glucose deprived (cOGD) conditions and a transient cerebral ischemia model mouse induced by transient middle cerebral artery occlusion (tMCAO) as a model for in vivo neurodegeneration and investigated the regulatory mechanism behind the expression of *Cfl1*.

## 2. Materials and Methods

### 2.1. Cell Culture and hnRNP Q1 KO Cells

Neuro2A (n2a) cells were cultured in Dulbecco’s modified Eagle’s medium (DMEM) (Hyclone) with 10% fetal bovine serum (FBS) (Hyclone) and 1% penicillin–streptomycin (Welgene). The cells were cultured in a CO_2_ incubator at 37 °C. HnRNP Q1 KO cells were previously generated in our laboratory [14]. Briefly, single-guide RNA (sgRNA) was designed through an online CRISPR design tool at http://crispr.mit.edu, accessed on 20 July 2019. Then, the double-stranded DNA of our target (hnRNP Q1) was cloned into the pSpCas9(BB)-2A-Puro (PX459) plasmid (Addgene, cat. 62988). PX459 plasmid were then transfected into Neuro2A cells, which were later isolated through puromycin selection (2 μg/mL; Sigma-Aldrich). HnRNP Q1 KO cells were also cultured in DMEM with 10% FBS and 1% penicillin–streptomycin in a CO_2_ incubator at 37 °C.

### 2.2. Plasmid Construction

The plasmids that were used in this study were generated previously by our lab [13]. Briefly, the PCR product of 5′UTR of mouse *Cfl1* (Primers: forward: ACGCGTCGACGCCGGAAGGCCGCCCCG; reverse: TCCCCCCGGGGTTTCCGGAAACGAAAGGGAGAC) was cloned into the pRF bicistronic vector that contains coding sequences of renilla luciferase (Rluc) and firefly luciferase (Fluc). Plasmids with the deletion of the D1 region of *Cfl1* 5′UTR (pRF ΔD1) were also generated similarly. These plasmids were used to measure the translational activity of *Cfl1* 5′UTR. The Rluc and Fluc coding sequences of pRF vectors were then replaced with coding sequences of mCherry and eGFP fluorescent protein with a myristoylation signal to measure the translational activity of *Cfl1* 5′UTR in the primary hippocampal neuron. 5′UTR of Cfl1 mRNA was also cloned into pSK vectors for in vitro transcription.

### 2.3. RNA Interference

N2a cells were transfected with small interfering RNAs (siRNAs) or short hairpin RNAs (shRNA) by electroporation using NEON™ transfection system (Invitrogen, Waltham, MA, USA) or by Lipofectamine 2000 (Invitrogen) according to the manufacturer’s instruction. The siRNAs that were used in this study are as follows: si-control (Bioneer; 5′-CCUACGCCACCAAUUUCGU-3′), si-hnRNP Q (Bioneer; 5′-AGACAGUGAUCUCUCUCAUTT-3′), si-hnRNP Q1 (Bioneer; 5′-GAUCAGAAGAGGAAAGAAATT-3′), si-hnRNP A1 (Bioneer; 5′-GGACUGUAUUUGUGACUAA-3′), and si-nPTB, which was bought from Dharmacon (siGENOME SMARTpool Mouse Ptbp2 siRNA; M-049626). The siRNA sequences of hnRNP Q1 and hnRNP A1 were used to generate shRNA. The oligonucleotides of hnRNP Q1 and hnRNP A1 were annealed and inserted into pLentiLox3.7 (pLL3.7) lentiviral plasmid.

### 2.4. SDS-Polyacrylamide Gell Electrophoresis (PAGE) and Immunoblotting

A total of 20 or 30 μg of cell lysates (protein in cell lysates) were mixed with 5× sample buffer (0.6% 1M Tris, 50% Glycerol, 10% SDS, 0.5% 2-Mercaptoethanol, and 1% Bromophenol blue) to create loading samples. The samples were loaded onto the Western blot gel and were resolved in electrophoresis chambers (Bio-Rad, Hercules, CA, USA). Then, the proteins in the gel were transferred to nitrocellulose membranes (Pall Corporation, NY, USA) using the same power supply and transfer chamber (Bio-Rad). For immunoblotting, the membranes were incubated with the primary antibodies for 12 h at 4 °C followed by secondary antibody for 2 h at room temperature. The membranes were visualized with the LAS-4000 system (FUJIFILM, Tokyo, Japan) after treating the membrane with enhanced chemiluminescent (ECL) solution.

### 2.5. RNA Extraction and RT-qPCR

The RNA from harvested cells or brain tissues were extracted using TRI reagent according to the manufacturer’s instructions. Briefly, the cell or brain tissues were homogenized in the TRI reagent before adding chloroform totaling 1/5th of the original volume. Then, the samples were incubated at room temperature for 10 min before centrifugation at 15,000 rpm, at 4 °C, for 10 min. Then, the supernatant of the samples was moved to a fresh e-tube, and an equal volume of isopropanol was added to the sample. After incubating the samples in ice for 10 min, the samples were centrifuged at 15,000 rpm, at 4 °C, for 10 min. After removing isopropanol from the samples, RNA pellets were washed in ethanol and dissolved in DEPC-treated water. Isolated RNAs from the cell or brain tissue were reverse-transcribed with Improm-II reverse transcription system from Promega following the provider’s instructions. The cDNA from RT-PCR was used to measure the RNA level of cells or brain tissue. FastStart Universal SYBR Green Master from Roche was used for the reaction while StepOnePlus Real-Time system was used to measure the level. Different primers of the target genes were used as follows: *Cfl1* (mouse), 5′-GCCAACTTCTAACCACAATAG-3′ and 5′-CCTTACTGGTCCTGCTTCC-3′; *Gapdh* (mouse), 5′-AAATGGTGAAGGTCGGTGTG-3′ and 5′-TGAAGGGGTCGTTGATGG-3′; *Rpl32* (mouse), 5′-AACCCAGAGGCATTGACAAC-3′ and 5′-CACCTCCAGCTCCTTGACAT-3′; *Tbp* (mouse), 5′-CAGCCTTCCACCTTATGCTC-3′ and 5′-TTGCTGCTGCTGTCTTTGTT-3′; *Hnrnpa1* (mouse), 5′-CTGTCGAAGCAAGAGATGGC-3′ and 5′-GCCTCCTCCATAACCACCAT-3′; *Syncrip* (mouse), 5′-ACCACCTCCAGATTCCGTTT-3′ and 5′-GCCTCTTGTGCTGCTTCTTT-3′; *Hif1α* (mouse), 5′-CTATGGAGGCCAGAAGAGGGTAT-3′, and 5′-CCCACATCAGGTGGCTCATAA-3′.

### 2.6. Luciferase Assay

Neuro2a cells were co-transfected with pRF vectors and siRNAs and harvested 24 h after transfection. Cells were lysed with the reporter lysis buffer (Promega, Madison, WI, USA), incubated in ice for 10 min and centrifuged at 15,000 rpm, at 4 °C, for 10 min before measuring the activity of luciferase of renilla or firefly. The luciferase activity was measured using Dual-Luciferase Reporter Assay system (Promega) as instructed by the manufacturer.

### 2.7. mRNA Stability Assay

To measure the stability of an mRNA, n2a cells were treated with actinomycin D (Sigma-Aldrich, St. Louis, MO, USA, A9415) (5 μg/mL), a transcription blocker, for the indicated times. Then, the RNAs were extracted from the cell as explained previously. The mRNA stability was measured through RT-qPCR.

### 2.8. RNA Immunoprecipitation

The n2a cells were lysed with RNA-IP buffer (10 mM HEPES (pH 7.5), 100 mM of KCl, 5 mM of MgCl2, 0.1% NP-40, protease inhibitor) and the lysates were incubated with mouse IgG, anti-hnRNP Q, or anti-hnRNP A1 at 4 °C for overnight. Then, the mixture was further incubated with Protein G agarose bead (Thermo Fisher Scientific, Waltham, MA, USA) for 4 to 6 h. The beads were washed three times before isolating the protein-bound RNAs using TRI reagent. The level of RNAs that were bound to the protein were quantified by RT-qPCR as explained previously.

### 2.9. In Vitro RNA Binding Assay

pSK vectors that contain Cfl1 5′UTR were linearized with Xba I restriction enzyme. The linearized vectors were in vitro transcribed using T7 RNA polymerase (Promega) in the presence of biotin-uridine 5′-triphosphate (UTP). The biotinylated RNA transcript of D1 region or D2 region of *Cfl1* 5′UTR (*Cfl1* 5′UTR D1 or *Cfl1* 5′UTR D2) was generated by Bioneer (Daejeon, Korea). The biotinylated RNA transcripts of *Cfl1* 5′UTR were incubated with normal n2a cell lysates or siRNA-transfected n2a cell lysates and were further subjected to incubation with streptavidin agarose beads (Thermo Fischer Scientific). The proteins that were bound on streptavidin agarose beads were pulled down and analyzed by SDS-PAGE followed by immunoblotting.

### 2.10. Primary Hippocampal Neuron Culture

Primary hippocampal neuron was prepared from E18 mouse embryos. The hippocampi were dissected and isolated from mouse embryo, which were later dissociated with trypsin (Sigma-Aldrich) and deoxyribonuclease I (Sigma-Aldrich). The dissociated tissues were plated onto poly-L-lysine-coated (Sigma-Aldrich) dishes for biochemical analysis or onto a poly-L-lysine-coated microscope cover glass for image analysis. Primary hippocampal neurons were cultured and maintained with Neurobasal media (Gibco, Waltham, MA, USA) supplemented with B27 (Gibco) and GlutaMAX-I (Gibco) in a humidified CO_2_ chamber (5%) at 37 °C. The media was exchanged with fresh media every 3 days. Different DNA vectors or siRNAs were transfected with Lipofectamine^TM^ 2000 (Invitrogen) at DIV 1 or DIV 2. The chemically oxygen–glucose-deprived (cOGD) neuron was induced between DIV 5~7 as described previously [7]. Briefly, the Neurobasal media that the neurons were cultured in was exchanged with balanced salt solution (BSS) (1.2 mM of CaCl_2_, 0.4 mM of MgSO_4_, 5.3 mM of KCl, 0.4 mM of KH_2_PO_4_, 137 mM of NaCl_2_, 0.3 mM of NaHPO_4_, 5 mM of glucose, and 10 mM of 1,4-piperazinediethanesulfonate (PIPES) buffer, pH 7.3.) or BSS with 6 mM of 2-deoxyglucose and 10 mM sodium azide (cOGD BSS). After 20 or 40 min of incubation at 37 °C, the control BSS or cOGD BSS were replaced with fresh control BSS and were further incubated at 37 °C for 10 min. Then, the control neuron or cOGD neuron were subjected to different experiments such as Western blot, immunocytochemistry, or fluorescent in situ hybridization. To minimize the effect of glial cells, the cultured plate was treated with 5 µM of cytosine arabinoside (AraC).

### 2.11. Immunocytochemistry

After siRNA transfection, shRNA transfection, or culturing in cOGD condition, mouse hippocampal neurons were fixed in 4% paraformaldehyde (PFA)–PBS solution for 20 min at room temperature. After several washes with PBS, the hippocampal neurons were permeabilized with 0.5% Triton X-100 dissolved in PBS for 10 min at room temperature, blocked with 5% FBS-PBS solution for 2 h and incubated with primary antibodies for 12 h at 4 °C. Then, the neurons were washed in PBS several times before they were incubated in Alexa Fluor-conjugated secondary antibodies for 1 h at room temperature. Neurons were then mounted with the fluorescent mounting medium (Dako) and were further observed with fluorescent microscope. The “*n*” represents the number of independent experiments.

### 2.12. Fractionation

To divide the nuclear fraction and cytoplasmic fraction of n2a cell or primary hippocampal neuron, the harvested cells or neurons were re-suspended in fractionation buffer (10 mM of HEPES, 10 mM of KCl, and 0.05% Np-40; pH 7.4). After 20 min of incubation in ice, re-suspended cells or neurons were centrifuged at 15,000 rpm at 4 °C for 10 min. After centrifugation, the cells or neurons are divided into supernatants and pellets. The supernatant was moved to a fresh e-tube and was used to analyze the cytoplasmic fraction of the cells and neurons. The pellet was re-suspended in radioimmunoprecipitation assay (RIPA) buffer and was used to analyze the nuclear fraction of the cells and neurons. Both the cytoplasmic fraction and nuclear fraction of cell lysates were further resolved by SDS-PAGE, immunoblotted with different antibodies, and visualized with the LAS-4000 system. To isolate the axonal fraction of neuron from the soma fraction, we cultured hippocampal neuron on a hanging insert (SPL) with filters (pore size 3.0 µm). Then, the top side of the filters were cleaned with cotton swab to remove the soma of the neuron. The filters were then isolated by cutting around the circumference of the hanging insert, and they were lysed with lysis buffer (axonal fraction). The samples were further resolved by SDS-PAGE, immunoblotted with different antibodies, and visualized with the LAS-4000 system.

### 2.13. RNA Fluorescence In Situ Hybridization (FISH)

RNA FISH was performed with Stellaris^®^ RNA FISH products from Biosearch Technologies (Hoddesdon, UK) according to the manufacturer’s instructions. Briefly, after the incubation with secondary antibodies from immunocytochemistry, the primary hippocampal neurons were washed several times with PBS. Then, the neurons were fixed in 4% PFA once again for 20 min at room temperature. Then, the neurons are washed with Wash Buffer A (Biosearch Technologies) for 5 min at room temperature, after several washes with PBS. While washing, custom probes that target *Cfl1* mRNA (Biosearch Technologies) and Hybridization Buffer (HB; Biosearch Technologies) were mixed in the ratio noted by the instructions. After the wash, the neurons are incubated with the probe–HB solution for 4 h at 37 °C. Then, the neurons were washed with Wash Buffer A for 30 min and with Wash Buffer B (Biosearch Technologies) for an additional 5 min at room temperature, before being mounted on the slide glass. “*n*” represents the number of independent experiments.

### 2.14. Mice and Transient Middle Cerebral Artery Occlusion (tMCAO)

Mice that were used in this study was used with protocols approved by the Institutional Animal Care and Use Committees of Sookmyung Women’s University. Transient middle cerebral artery occlusion (tMCAO) was performed to induce the transient cerebral ischemia model mouse. Before the MCA occlusion, a mouse was first narcotized with 2.0% isoflurane (VS Pharm, Hanam, Korea) in 30% oxygen and 70% nitrous oxide using a facemask. Every mouse was placed on a heating pad during the entire surgical procedure to maintain the body temperature. Then, the midline of neck of the mouse was incised to expose the left common carotid artery (CCA), internal carotid artery (ICA), and external carotid artery (ECA). After clipping the CCA, two knots were made on either side of the ECA. After cutting in between the two knots of the ECA, nylon monofilament suture (Filament size 6–0, diameter 0.09–0.11 mm, length 20 mm; Doccol Corporation, Sharon, MA, USA) was inserted into the lumen of ECA, which was further advanced to the lumen of ICA, to block the blood flow. After 45 min of blocking the blood flow by tMCAO, the mouse brain was re-perfused as the nylon suture was removed. Two knots of the ECA were closed, and the clip was removed from CCA. The midline of the neck of the mouse was closed by stitching. The sham mice were surgically operated on in a similar way except for the cutting of the ECA and insertion of nylon suture. The mice were sacrificed 24 h after the tMCAO surgery.

### 2.15. Immunohistochemistry

The brains of tMCAO mice were isolated and fixed with 4% PFA-PBS solution. Then, the brains were dehydrated through a sequential wash with different percentages of ethanol before they were frozen with O.C.T. compound Tissue-Tek (Sakura, Torrance, CA, USA, 4583). The 30-micrometer-thick coronal sections of frozen tMCAO brains were collected using a cryostat (Leica CM1850). The brain sections were washed three times with PBS and blocked with blocking buffer (5% Fetal Bovine serum, 3% bovine serum albumin, 0.3% Triton X-100 in PBS) for 1 h at room temperature. Then, the brain sections were incubated with primary antibodies that were dissolved in blocking buffer for 12 h at 4 °C. After several washes with PBS, the brain sections were incubated with Alexa Fluor™ (Invitrogen)-conjugated secondary antibodies for 1 h at room temperature. Then, the brain sections were stained with Hoechst (5 μg/mL) for 10 min after several washes with PBS. The brain sections were then mounted with the fluorescence mounting medium (Dako, Thermo Fischer) before imaging with Zeiss LSM 800 Epifluorescence microscope. “*n*” represents the number of mice.

### 2.16. Imaging

For immunocytochemistry analysis and RNA FISH, FV1000 Confocal microscope (Olympus) or FV3000 Confocal Laser Scanning microscope (Olympus, Tokyo, Japan) was used. The fluorescent images were obtained using Coherent^®^ High Performance OBIS™ laser with wavelengths of 405 nm, 488 nm, and 561 nm. The z-stack images were also taken using the same imaging system (11~12 stacks, 0.480 μm/slices, Olympus). The images were exported using the FV31S-SW program. For immunohistochemistry analysis, the fluorescent signals were detected with Zeiss LSM 800 Epifluorescence microscope with excitation and emission wavelengths of 358/461 nm, 495/519 nm, and 590/617 nm. The intensity or co-localization of fluorescent signals of immunocytochemistry images or immunohistochemistry images were analyzed using ImageJ (NIH, Bethesda, MD, USA).

### 2.17. Antibody

For primary antibodies in Western blot analysis, Anti-hnRNP Q (1:1000; Sigma-Aldrich, R5653), Anti-hnRNP A1 (1:1000; Santa Cruz, sc-32301), Anti-Cofilin (1:250; Abcam, ab54532, ab42824), Anti-14-3-3ζ (1:1000; Santa Cruz, sc-1657, sc-1019), Anti-GAPDH (1:1000; Bethyl, A300-641A), normal Anti-Mouse IgG (1:1000; Santa Cruz, sc-2025), Anti-nPTB (1:1000; Abcam, ab154787), Anti-Lamin B (1:1000; Santa Cruz, sc-6216), Anti-RNA PolII (1:1000; Abcam, ab5408), and Anti-NeuN (1:1000; Sigma-Aldrich, MAB377) were used. For secondary antibodies in Western blot analysis, horseradish peroxidase (HRP)-conjugated Anti-Mouse IgG (1:10,000; Invitrogen, 31430), HRP-conjugated Anti-Rat IgG (1:10,000; Bethyl, A110-105P), HRP-conjugated Anti-Goat IgG (1:10,000; Bethyl, A50-101P), and HRP-conjugated Anti-Rabbit IgG (1:10,000; Promega, W4018) were used. For primary antibodies in immunocytochemistry and immunohistochemistry analysis, Anti-Cofilin (1:100; Abcam, ab54532, ab42824), Anti-hnRNP Q (1:200; Sigma-Aldrich, R5653), Anti-hnRNP A1 (1:200; Santa Cruz, sc-32301), Anti-MAP2 (1:500; Abcam, ab5392), Anti-Tau (1:300; Abcam, ab64193), and Anti-NeuN (1:100; Sigma-Aldrich, MAB377) were used. For secondary antibodies in immunocytochemistry and immunohistochemistry analysis, Anti-Mouse IgG conjugated with Alexa Fluor™ 488 (1:1000; Invitrogen, A-11,001), Alexa Fluor™ 405 (1:1000; Invitrogen, A-31553), or Alexa Fluor™ 594 (1:1000; Invitrogen, A-11005) was used. Additionally, Anti-Rabbit IgG conjugated with Alexa Fluor™ 488 (1:1000; Invitrogen, A-11008), Alexa Fluor™ 405 (1:1000; Invitrogen, A-31556), or Alexa Fluor™ 594 (1:1000; Invitrogen, A-11012) and Anti-Chicken IgY conjugated with Alexa Fluor™ 488 (1:1000; Abcam, ab150169). To detect cofilin–actin rods in primary hippocampal neurons, Alexa Fluor™ 488-conjugated phalloidin (Invitrogen, A12379) was used to stain filamentous actin (F-Actin) according to the manufacturer’s instructions.

### 2.18. Experimental Design and Statistical Analysis

All cell-based data are the results of at least three independent experiments performed with cells from different passage. All microscopy experiments with primary hippocampal neurons were repeated more than 3 times with neurons from different biological samples (mice). The comparison between the two groups were statistically analyzed by unpaired Student’s *t* tests. Comparisons between three or more groups with one independent variable were analyzed by ordinary one-way analysis of variance (ANOVA) with Tukey’s multiple comparison test. When there were two independent variables in the experiment, ordinary two-way ANOVA with Tukey’s multiple comparison test was used for analysis. All quantitative data are presented as means ± SD. *p* values greater than 0.05 were not considered significant. The significance of the statistical analysis was indicated as such: n.s., not significant, * *p* ≤ 0.05, ** *p* ≤ 0.01, *** *p* ≤ 0.001, and **** *p* ≤ 0.0001.

## 3. Results

### 3.1. The Translational Activity of Cfl1 mRNA Is Increased in cOGD Neurons

**Figure 1:**
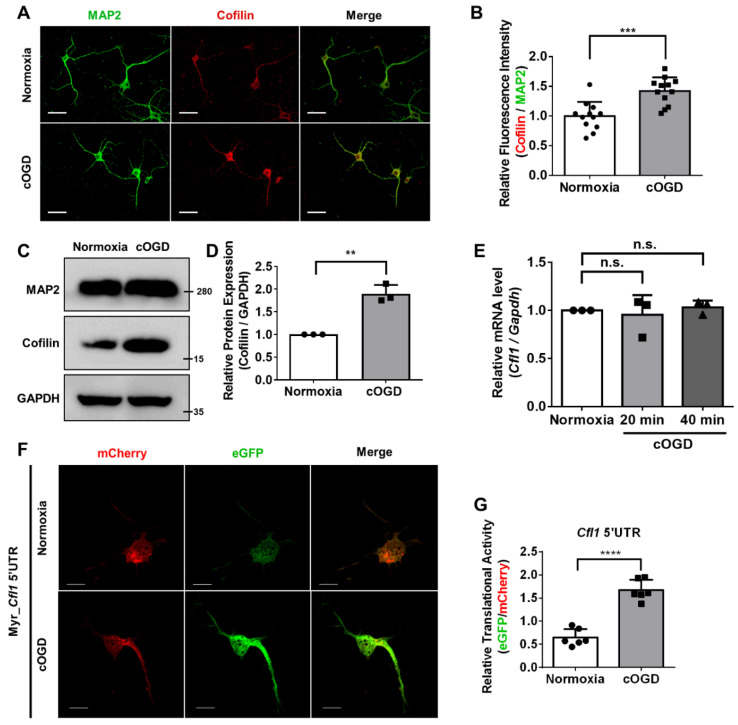
The translational activity of *Cfl1* mRNA is enhanced in cOGD neuron. (**A**) The immunofluorescence labeling of cofilin (red) and MAP2 (green) in the primary hippocampal neuron that was cultured under normoxia conditions (top) or chemically oxygen–glucose-deprived (cOGD) conditions (bottom). Scale bar = 30 μM. (**B**) Quantification of relative fluorescence intensity of cofilin from (**A**) normalized to MAP2 (*n* = 12). (**C**) Representative immunoblot of cofilin in normal neuron (left) and cOGD neuron (right). GAPDH was used as loading control. (**D**) Quantification of protein level of cofilin from (**C**). ImageJ was used to measure the intensity of the blot (*n* = 3). (**E**) Quantitative reverse transcription polymerase chain reaction (RT-qPCR) analysis of *Cfl1* mRNA level in primary hippocampal neuron cultured under normoxia conditions (white) and cOGD conditions (grays) (*n* = 3). (**F**) Measuring the translational activity of *Cfl1* mRNA using mCherry (red)—eGFP (green) bicistronic vector with *Cfl1* 5′UTR, in the primary hippocampal neuron that was cultured under normoxia conditions (top) and cOGD conditions (bottom). Scale bar = 10 μM. (**G**) Quantification of relative translational activity of *Cfl1* mRNA by measuring the eGFP to mCherry ratio in the normal neuron (white) and cOGD neuron (gray) (*n* = 6). n.s., not significant, ** *p* ≤ 0.01, *** *p* ≤ 0.001, **** *p* ≤ 0.0001; unpaired Student’s *t* test was performed for (**B**,**D**,**G**); ordinary one-way ANOVA with Tukey’s multiple comparison test was performed for (**E**). “*n*” represents the number of independent experiments. Error bars indicate SDs.

Since previous studies collectively showed the relationship between the expression of *Cfl1* and cofilin–actin aggregates formation, we first checked the protein level of cofilin in neurons under OGD conditions. We cultured primary hippocampal neurons from E18 mouse embryos under cOGD conditions to mimic neurons that are degenerating due to OGD and those under normoxia conditions for comparison [7]. Immunofluorescence staining (Figure 1A,B) and Western blot (Figure 1C,D) results show that the expression cofilin was increased in a cOGD neuron, although quantitative reverse-transcription polymerase chain reaction (RT-qPCR) results show the unaltered mRNA level of *Cfl1* (Figure 1E). This shows that the increase in protein level of cofilin in the cOGD neuron was not influenced by the transcription of *Cfl1*.

Then, we investigated the translation of *Cfl1* mRNA in cOGD neurons. We transfected cOGD neurons with bicistronic vectors that contain coding sequences of mCherry and eGFP (Figure S1A). Since we previously revealed the IRES element in 5′untranslated region (5′UTR) of *Cfl1* mRNA [13], we inserted *Cfl1* 5′UTR between the coding sequences of two fluorescent proteins (Figure S1A). The ratio of eGFP to mCherry represents the translational activity of *Cfl1* 5′UTR (Figure S1B). Interestingly, the translational activity of *Cfl1* 5′UTR was significantly upregulated in the cOGD neuron (Figure 1F,G). These results show that the increased protein level of cofilin under OGD conditions may have been induced by the upregulated translation of *Cfl1* mRNA.

### 3.2. HnRNP Q and hnRNP A1 Regulate the Translational Activity of Cfl1 mRNA

The translational activity of an mRNA is regulated by different factors, such as RNA-binding proteins or micro-RNAs (miRNA) [15,16,17]. We previously demonstrated that an RNA-binding protein, neural polypyrimidine tract-binding proteins (PTBP2), could promote the translation of *Cfl1* mRNA [13]. Thus, to investigate whether other RNA-binding proteins had a similar function, we used an Orbitrap on *Cfl1* 5′UTR (Figure S2A). The Orbitrap result shows that many RNA-binding proteins, such as heterogeneous nuclear ribonucleoproteins (hnRNP), were bound onto *Cfl1* mRNA (Figure S2A). Since hnRNP family proteins are known to be associated with neurodegenerative diseases [14,18], we hypothesized that hnRNP Q and hnRNP A1 could regulate the translation of *Cfl1* mRNA.

**Figure 2:**
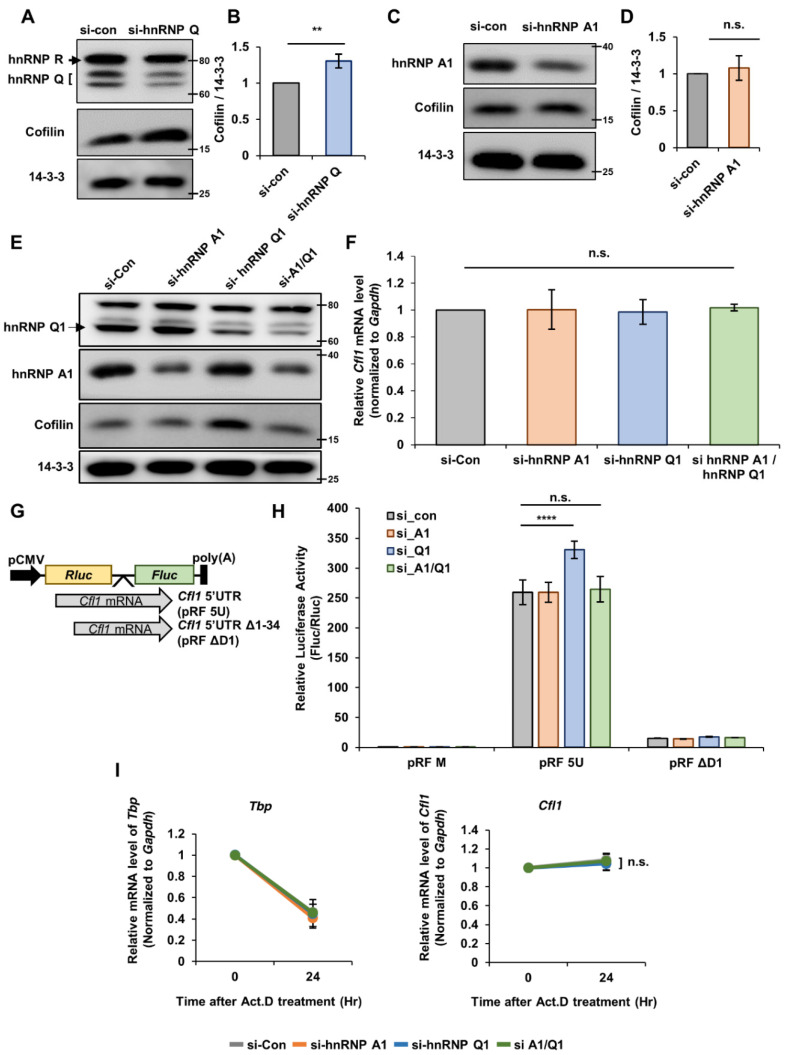
HnRNP Q and hnRNP A1 regulates the translational activity of *Cfl1* mRNA. (**A**,**B**) Representative immunoblot (**A**) and quantification (**B**) of hnRNP Q knockdown experiment. N2a cells were transfected with si-control (left lane (**A**); gray (**B**)) or si-hnRNP Q (right lane (**A**); blue (**B**)). Immunoblot of 14-3-3ζ was used for normalization. ImageJ was used to measure the intensity of the blot (*n* = 3). (**C**,**D**) Representative immunoblot (**C**) and quantification (**D**) of hnRNP A1 knockdown experiment. N2a cells were transfected with si-control (left lane (**C**); gray (**D**)) or si-hnRNP A1 (right lane (**C**); orange (**D**)). 14-3-3ζ was used for normalization. ImageJ was used to measure the intensity of the blot (*n* = 3). (**E**) Representative immunoblot of double (hnRNP Q1 and hnRNP A1) knockdown experiment. N2a cells were transfected with si-control (first lane), si-hnRNP A1 (second lane), si-hnRNP Q1 (third lane), or both siRNAs (fourth lane). 14-3-3ζ was used as a loading control. (**F**) RT-qPCR analysis of *Cfl1* mRNA level in n2a cells transfected with si-control (gray), si-hnRNP A1 (orange), si-hnRNP Q1 (blue), or both siRNAs (green). *Gapdh* mRNA was used for normalization (*n* = 3). (**G**) Illustration of bicistronic vector that we used to measure the translational activity of *Cfl1* mRNA 5′UTR. 5′UTR sequences of *Cfl1* mRNA (gray) were inserted in between the sequences of *Rluc* (yellow) and *Fluc* (green). (**H**) Measurement of translational activity of *Cfl1* 5′UTR in n2a cells transfected with si-control (gray), si-hnRNP A1 (orange), si-hnRNP Q1 (blue), or both siRNAs (green). Relative luciferase activity of Fluc and Rluc measured by dual-luciferase assay indicates translational activity of *Cfl1* 5′UTR (*n* = 3). (**I**) Measuring the mRNA stability of *Tbp* mRNA (left) or *Cfl1* mRNA (right) in n2a cells treated with actinomycin D (5 μg/mL) after the transfection of si-control (gray), si-hnRNP A1 (orange), si-hnRNP Q1 (blue), or both siRNAs (green) through RT-qPCR. *Tbp* mRNA was used as the experimental control, and *Gapdh* mRNA was used for normalization (*n* = 3). n.s., not significant, ** *p* ≤ 0.01, **** *p* ≤ 0.0001; unpaired Student’s *t* test was performed for (**B**,**D**); ordinary one-way ANOVA with Tukey’s multiple comparison test was performed for (**F**); two-way ANOVA with Tukey’s multiple comparison test was performed for (**H**,**I**). Error bars indicate SDs.

To address our hypothesis, we first checked whether hnRNP Q or hnRNP A1 affects the level of cofilin in neuro2a (n2a) cells. When we knocked down hnRNP Q through siRNA transfection, the level of cofilin significantly increased (Figure 2A,B). Additionally, there are three isoforms to hnRNP Q (hnRNP Q1, Q2 and Q3) [19], but hnRNP Q1 had a major effect on the level of cofilin since the knockdown of hnRNP Q1 alone was enough to increase the level of cofilin (Figure S2B). Additionally, the primary hippocampal neuron had greatest amount of hnRNP Q1 out of the three isoforms (Figure S2C), so we continued our experiments with focuses on hnRNP Q1 in finding the role of hnRNP Q. With hnRNP A1, the knockdown of hnRNP A1 did not change the level of cofilin (Figure 2C,D). Interestingly, however, when we knocked down both hnRNP Q and hnRNP A1, the increased level of cofilin was no longer observable (Figure 2E and Figure S2D). These data imply that hnRNP Q and hnRNP A1 may simultaneously regulate the level of cofilin.

Although we knocked down hnRNP Q and hnRNP A1 to confirm their role in *Cfl1* transcription, the level of *Cfl1* mRNA was unaffected (Figure 2F). Then, to determing if hnRNP Q and hnRNP A1 participates *Cfl1* mRNA translation, we transfected the n2a cells with bicistronic vectors that contain renilla luciferase (*Rluc*) and firefly luciferase (*Fluc*) sequences with *Cfl1* 5′UTR between two coding sequences (Figure 2G). The ratio of Fluc to Rluc luciferase activity represents the relative translational activity of *Cfl1* mRNA. Similar to the protein level, the knockdown of hnRNP A1 did not affect the translational activity of *Cfl1* mRNA, while the knockdown of hnRNP Q significantly upregulated the translational activity of *Cfl1* mRNA (Figure 2H). Additionally, the double knockdown of hnRNP Q and hnRNP A1 alleviated the increased translation of *Cfl1* mRNA (Figure 2H). This indicates that hnRNP Q has a negative effect on the translation of *Cfl1* mRNA but interacts with hnRNP A1 in such a process.

RNA-binding proteins can indirectly affect the translation of an mRNA by regulating their stability [20]. Hence, we checked the stability of *Cfl1* mRNA by treating actinomycin D (Act.D), a transcription inhibitor. Interestingly, while the mRNA level of *TATA-binding protein* (*Tbp*) was substantially decreased by the actinomycin D treatment, the mRNA level of *Cfl1* was unaltered by the drug treatment (Figure 2I). Additionally, the knockdown of hnRNP Q or hnRNP A1, which was confirmed through RT-qPCR (Figure S2E), did not alter the stability of *Cfl1* mRNA (Figure 2I). Altogether, these data imply that hnRNP Q and hnRNP A1 simultaneously regulate the translation of *Cfl1* mRNA and not its stability.

### 3.3. HnRNP Q Inhibits the Interaction between hnRNP A1 and Cfl1 mRNA

**Figure 3:**
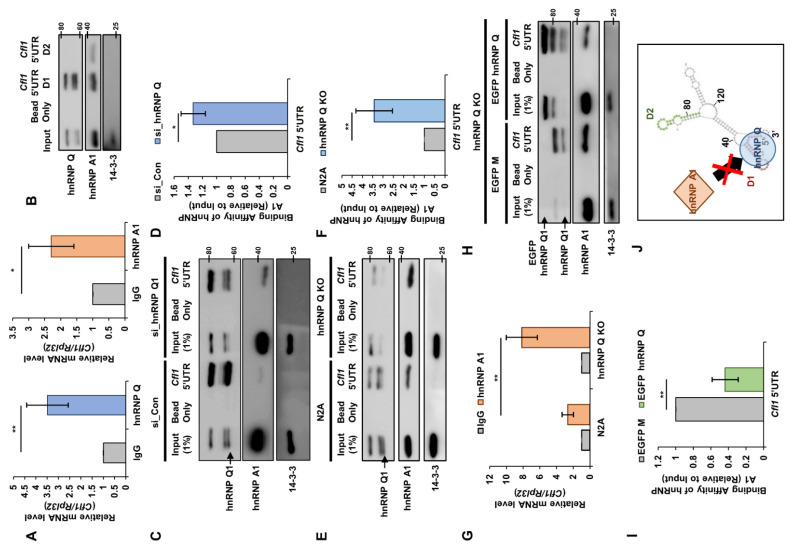
HnRNP Q interrupts the interaction between hnRNP A1 and *Cfl1* mRNA. (**A**) RT-qPCR analysis of *Cfl1* mRNA that interacts with either hnRNP Q (left) or hnRNP A1 (right). Immunoprecipitation was performed using mouse IgG antibody (gray), hnRNP Q antibody (blue), or hnRNP A1 antibody (orange). *Rpl32* mRNA was used for normalization (*n* = 3). (**B**) Representative immunoblot of in vitro RNA binding assay with biotinylated oligomer of D1 region of *Cfl1* 5′UTR (*Cfl1* 5′UTR D1) or D2 region of *Cfl1* 5′UTR (*Cfl1* 5′UTR D2) to measure the level of hnRNP Q or hnRNP A1 that was bound to each oligomer. Each oligomer was incubated in n2a cell lysates and pulled down with streptavidin. (**C**,**D**) Representative immunoblot (**C**) and the quantification (**D**) of binding affinity between hnRNP A1 and *Cfl1* mRNA during the knockdown of hnRNP Q. In vitro transcribed and biotinylated *Cfl1* 5′UTR was incubated in the lysates of n2a cells that were transfected with si-control (left three lanes (**C**); gray (**D**)) or si-hnRNP Q1 (right three lanes (**C**); blue (**D**)) and pulled down with streptavidin. The interaction was normalized to the hnRNP A1 of the input (first lane), which was normalized by 14-3-3ζ. ImageJ was used to measure the intensity of the blot (*n* = 3). (**E**,**F**) Representative immunoblot (**E**) and the quantification (**F**) of binding affinity between hnRNP A1 and *Cfl1* mRNA in hnRNP Q1 KO cells. In vitro transcribed and biotinylated *Cfl1* 5′UTR was incubated in the lysates of n2a cells (left three lanes (**E**); gray (**F**)) or hnRNP Q1 KO cells (right three lanes (**E**); blue (**F**)) and pulled down with streptavidin. The interaction was normalized to the hnRNP A1 of the input (first lane) which was normalized by 14-3-3ζ. ImageJ was used to measure the intensity of the blot (*n* = 3). (**G**) RT-qPCR analysis of *Cfl1* mRNA that interacts with either mouse IgG (gray) or hnRNP A1 (orange) in n2a cells (left) or hnRNP Q1 KO cells (right). Immunoprecipitation was performed using mouse IgG antibody or hnRNP A1 antibody. *Rpl32* mRNA was used for normalization (*n* = 3). (**H**,**I**) Representative immunoblot (**H**) and the quantification (**I**) of binding affinity between hnRNP A1 and *Cfl1* mRNA in hnRNP Q1 rescued hnRNP Q1 KO cells. In vitro transcribed and biotinylated *Cfl1* 5′UTR was incubated in the lysates of hnRNP Q1 KO cells transfected with EGFP-Mock (left three lanes (**H**); gray (**I**)) or EGFP-hnRNP Q1 (right three lanes (**H**); green (**I**)) and pulled down with streptavidin. The interaction was normalized to the hnRNP A1 of the input (first lane), which was normalized by 14-3-3ζ. ImageJ was used to measure the intensity of the blot (*n* = 3). (**J**) Illustration of regulatory mechanism of *Cfl1* mRNA translation by hnRNP Q1 and hnRNP A1. HnRNP Q1 inhibits the interaction between hnRNP A1 and 5′UTR of *Cfl1* mRNA under normal conditions. * *p* ≤ 0.05, ** *p* ≤ 0.01; unpaired Student’s *t* test was performed for (**A**,**D**,**F**,**I**); two-way ANOVA with Sidak’s multiple comparison test was performed for (**G**). Error bars indicate SDs.

Although the Orbitrap results show that hnRNP Q and hnRNP A1 interact with *Cfl1* mRNA, we wanted to confirm whether they actually interacted with *Cfl1* mRNA. We performed RNA immunoprecipitation (IP) with hnRNP Q or hnRNP A1 antibodies (Figure S3A,B). We found that both hnRNP Q and hnRNP A1 interact with *Cfl1* mRNA significantly more than the mouse IgG control (Figure 3A). Then, we checked which regions of *Cfl1* mRNA these proteins interacted in. When we removed an important IRES element from *Cfl1* 5′UTR (pRF ΔD1) [13], the translational activity of *Cfl1* mRNA was completely diminished (Figure 2H), which indicates that hnRNP Q and hnRNP A1 may interact at the IRES element. Thus, we biotinylated the D1 region (*Cfl1* 5′UTR D1) or D2 region of *Cfl1* 5′UTR (*Cfl1* 5′UTR D2) and pulled them down using streptavidin beads (Figure S3C). Both hnRNP Q and hnRNP A1 were mainly bound to the D1 region of *Cfl1* 5′UTR (Figure 3B). This demonstrates that hnRNP Q and hnRNP A1 interact with the IRES element that is crucial to t*Cfl1* mRNA translation.

In a cell, multiple proteins may simultaneously regulate the metabolism of an RNA [17,21]. As such, we observed that the effect of hnRNP Q on *Cfl1* mRNA translation requires the presence of hnRNP A1 (Figure 2H). This result implies that hnRNP Q and hnRNP A1 may function as trans-acting factors that simultaneously regulate the translation of *Cfl1* mRNA. Since both proteins interact at the D1 region of *Cfl1* 5′UTR, we speculated that their binding affinity may change with the presence of the other. Hence, we reduced the level of hnRNP Q or hnRNP A1 and measured their binding affinity to *Cfl1* 5′UTR. When we reduced the amount of hnRNP Q, the interaction between hnRNP A1 and *Cfl1* 5′UTR was significantly increased (Figure 3C,D). Additionally, reducing the level of all isoforms of hnRNP Q did not increase such interaction further, which confirms that hnRNP Q1 has the stronger effect (Figure S3D). On the other hand, reducing the level of hnRNP A1 did not alter the interaction between hnRNP Q and *Cfl1* 5′UTR (Figure S3E,F). These results suggest that hnRNP Q has a higher binding affinity toward *Cfl1* 5′UTR and potentially inhibits the interaction between hnRNP A1 and *Cfl1* 5′UTR.

To confirm, we used hnRNP Q knock-out (KO) cells that were previously modified in our laboratory [14]. We noticed that the interaction between hnRNP A1 and *Cfl1* 5′UTR was significantly increased in hnRNP Q KO cells (Figure 3E,F). RNA IP with hnRNP A1 antibody (Figure S3G) showed similar results since the level of *Cfl1* mRNA was significantly higher in hnRNP Q KO cells (Figure 3G). Intriguingly, the interaction between hnRNP A1 and *Cfl1* 5′UTR was substantially decreased when we restored the level of hnRNP Q (Figure 3H,I). This shows that hnRNP Q actually inhibits the interaction between hnRNP A1 and *Cfl1* 5′UTR, which leads to a negative effect on *Cfl1* mRNA translation.

We previously showed that nPTB interacts with the IRES element of *Cfl1* 5′UTR [13]. Since nPTB shares a binding location with hnRNP Q and hnRNP A1, there might be a possible interruption by nPTB. However, the interaction between nPTB and *Cfl1* 5′UTR was unaffected in hnRNP Q KO cells (Figure S4A,B). Additionally, when we knocked down nPTB (Figure S4C), there was no change in the interaction between hnRNP Q or hnRNP A1 and *Cfl1* 5′UTR (Figure S4D). This suggests that the translational regulation by hnRNP Q and hnRNP A1 is independent from nPTB. Overall, these results suggest that, in the presence of both proteins, hnRNP Q inhibits the interaction between hnRNP A1 and *Cfl1* 5′UTR, reducing the translational activity (Figure 3J).

### 3.4. The Level of Cofilin and the Translational Activity of Cfl1 mRNA in Primary Hippocampal Neuron Are Unaffected by hnRNP Q and hnRNP A1 under Normal Conditions

**Figure 4:**
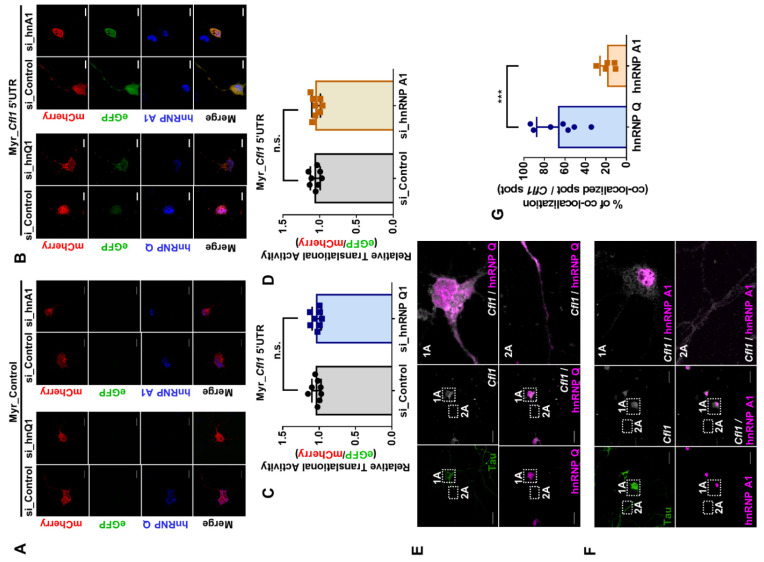
The translational activity of *Cfl1* mRNA in primary hippocampal neurons is unaffected by hnRNP Q and hnRNP A1 under normal conditions. (**A**,**B**) Measurement of the translational activity of *Cfl1* mRNA in primary hippocampal neuron that was transfected with si_Control (first and third column), si_hnRNP Q1 (second column) or si_hnRNP A1 (fourth column), with co-transfection of mCherry (red)/eGFP (green) bicistronic vector with (**B**) or without (**A**) *Cfl1* 5′UTR sequences. HnRNP Q (blue) and hnRNP A1 (blue) were stained by their corresponding antibodies. Scale bar = 10 μM. (**C**) Quantification of relative translational activity of *Cfl1* mRNA in hippocampal neurons transfected with si_Control (gray) or si_hnRNP Q1 (blue) by measuring the eGFP/mCherry ratio. n.s., not significant; unpaired Student’s *t* test (*n* = 8). (**D**) Quantification of relative translational activity of *Cfl1* mRNA in hippocampal neurons transfected with si_Control (gray) or si_hnRNP A1 (orange), by measuring the eGFP/mCherry ratio. n.s., not significant; unpaired Student’s *t* test (*n* = 8). (**E**) The co-localization between *Cfl1* mRNA and hnRNP Q was observed by the immunofluorescence labeling of hnRNP Q (magenta) and Tau (green), along with RNA fluorescence in situ hybridization (RNA FISH) of *Cfl1* mRNA (white) using Stellaris™ RNA FISH. The co-localization of hnRNP Q and *Cfl1* mRNA in soma (1A) and axon (2A) of the neuron is shown through scaled-up images. Scale bar = 30 μM. (F) The co-localization between *Cfl1* mRNA and hnRNP A1 was observed by the immunofluorescence labeling of hnRNP A1 (magenta) and Tau (green), along with RNA fluorescence in situ hybridization (RNA FISH) of *Cfl1* mRNA (white) using Stellaris™ RNA FISH. The co-localization of hnRNP A1 and *Cfl1* mRNA in the soma (1A) and axon (2A) of the neurons is shown through scaled-up images. Scale bar = 30 μM. (**G**) Quantification of co-localization between *Cfl1* mRNA and hnRNP Q (blue) or hnRNP A1 (orange) from experiments in (**E**,**F**), measured by the percentage of co-localization (co-localized *Cfl1* mRNA spot/total *Cfl1* mRNA spot). *** *p* ≤ 0.001; unpaired Student’s *t* test (*n* = 7). “*n*” represents the number of independent experiments. Data are represented as ± SD.

A discrepancy often exists between the result from the immortalized cell line and the primary cells because the cell lines often do not represent the true characteristic of the actual tissue [22,23]. Thus, to observe the translational regulatory role of hnRNP Q and hnRNP A1 in a biologically relevant context, we cultured primary hippocampal neuron from E18 mouse embryos. We then knocked down hnRNP Q or hnRNP A1 using GFP-tagged short hairpin RNA (shRNA) and measured the level of cofilin. Surprisingly, although the level of hnRNP Q (Figure S5A–C) and hnRNP A1 (Figure S5D,F) was reduced in GFP-tagged neurons, the level of cofilin was unaffected. We also measured the translational activity of *Cfl1* mRNA in primary hippocampal neurons after reducing the level of hnRNP Q or hnRNP A1 (Figure 4A,B). Unlike the results from n2a cells, the translational activity of *Cfl1* mRNA was unaffected by the knockdown of hnRNP Q or hnRNP A1 (Figure 4C,D).

The difference in results between the n2a cells and primary hippocampal neurons might be due to a contrasting protein localization [24,25]. Hence, we examined the interaction between hnRNP Q or hnRNP A1 and *Cfl1* mRNA in primary hippocampal neurons through RNA fluorescence in situ hybridization (RNA FISH). About 65% of *Cfl1* mRNA spots co-localized with hnRNP Q, while only 17% of *Cfl1* mRNA spots co-localized with hnRNP A1 (Figure 4E–G). Additionally, the interaction with hnRNP Q was localized in soma and the axon (Figure 4E, box 1A and 2A), while the interaction with hnRNP A1 was found only in the nucleus (Figure 4F, box 1A and 2A). Although we previously observed that hnRNP Q inhibits the interaction between hnRNP A1 and *Cfl1* mRNA in n2a cells (Figure 3C–G), hnRNP Q could not inhibit such interaction in neurons since hnRNP A1 is mainly localized in the nucleus. This may be why the level of cofilin and translational activity of *Cfl1* mRNA was unaffected by the knockdown of hnRNP Q in primary hippocampal neurons.

**Figure 5:**
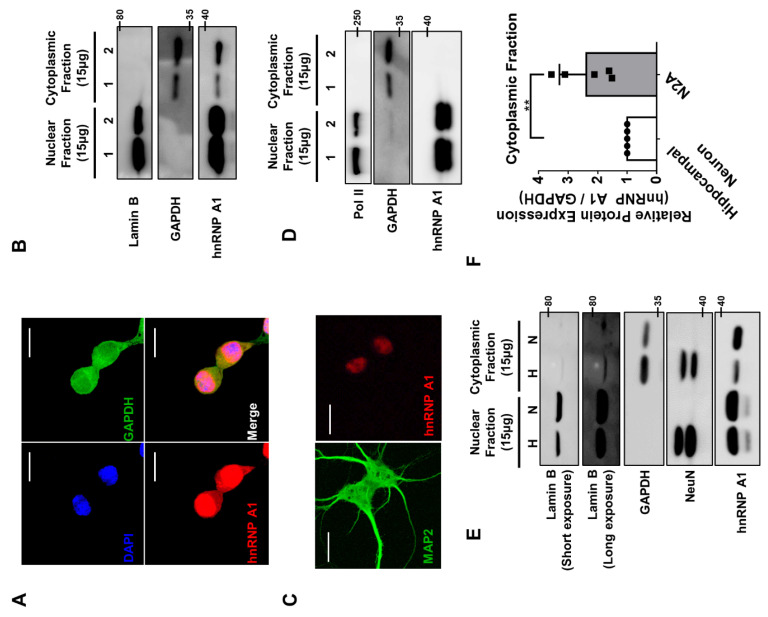
Different localization of hnRNP A1 in n2a cells and primary hippocampal neurons. (**A**) The Z-stack image of immunofluorescence staining of hnRNP A1 (red), GAPDH (green), and DAPI (blue) in n2a cells were obtained to observe the localization of hnRNP A1 in n2a cells. A total of 12 images with the interval of 0.480 μm/slices were stacked together. Scale bar = 20 μM. (**B**) Representative immunoblot of hnRNP A1 in n2a cells after nuclear fractionation. Lamin B was used as the loading control for nuclear fraction, while GAPDH was used as the loading control for cytoplasmic fraction. (**C**) Representative image of primary hippocampal neurons stained with hnRNP A1 (red) and MAP2 (green). Scale bar = 20 μM. (**D**) Representative immunoblot of hnRNP A1 in primary hippocampal neurons after nuclear fractionation. Pol II was used as the loading control for nuclear fraction, while GAPDH was used as the loading control for cytoplasmic fraction. (**E**) Representative immunoblot that compares the level of hnRNP A1 between n2a cells (indicated by N) and primary hippocampal neurons (indicated by H) after nuclear fractionation. Lamin B was used as the loading control for nuclear fraction, while GAPDH was used as the loading control for cytoplasmic fraction. NeuN was used as the marker for primary hippocampal neuron. (**F**) Quantification of protein level of hnRNP A1 in the cytoplasmic fraction of hippocampal neuron (white) and n2a cells (gray). GAPDH was used for normalization. ** *p* ≤ 0.01; unpaired Student’s *t* test (*n* = 5). Data are represented as ± SD.

Then, to confirm if there is a discrepancy between n2a cells and primary hippocampal neurons in regard to hnRNP A1, we looked at the localization of hnRNP A1. When we stained and immunoblotted hnRNP A1 in n2a cells, the protein was localized in both the nucleus and cytoplasm (Figure 5A,B). On the other hand, hnRNP A1 in primary hippocampal neurons was mainly located in the nucleus (Figure 5C,D). When we compared the level of hnRNP A1 in n2a cells and primary hippocampal neuron, n2a cells had significantly more hnRNP A1 in the cytoplasmic fraction than the neuron did, although there has been slight leakage of nuclear fraction (Figure 5E,F). These data indicate that translational activity of *Cfl1* mRNA in primary hippocampal neuron is unaffected by hnRNP Q and hnRNP A1 under normal conditions due to difference in the localization of hnRNP A1.

### 3.5. The Interaction between hnRNP A1 and Cfl1 mRNA Increases under cOGD Conditions Due to Re-Localization of hnRNP Q and hnRNP A1

**Figure 6:**
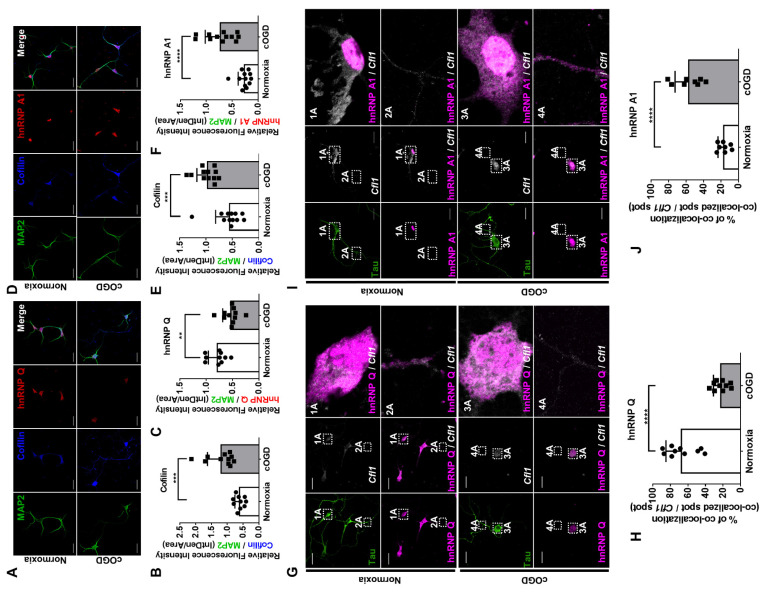
HnRNP Q and hnRNP A1 re-localizes under cOGD conditions, which increases the interaction between hnRNP A1 and *Cfl1* mRNA. (**A**) The immunofluorescence labeling of cofilin (blue), hnRNP Q (red), and MAP2 (green) in primary hippocampal neuron that was cultured under normoxia conditions (top) and cOGD conditions (bottom). Scale bar = 30 μM. (**B**,**C**) Quantification of relative fluorescence intensity of cofilin (**B**) and hnRNP Q (**C**) in normal hippocampal neuron (white) or cOGD neuron (gray), normalized to the intensity of MAP2 (*n* = 11). (**D**) The immunofluorescence labeling of cofilin (blue), hnRNP A1 (red), and MAP2 (green) in primary hippocampal neuron that was cultured under normoxia conditions (top) and cOGD conditions (bottom). Scale bar = 30 μM. (**E**,**F**) Quantification of relative fluorescence intensity of cofilin (**E**) and hnRNP A1 (**F**) in normal hippocampal neuron (white) and cOGD neuron (gray), normalized to the intensity of MAP2 (*n* = 12). (**G**) The co-localization between *Cfl1* mRNA and hnRNP Q in primary hippocampal neuron that was cultured under normoxia conditions (top) and cOGD conditions (bottom) was observed by the immunofluorescence labeling of hnRNP Q (magenta) and Tau (green), along with the RNA FISH of *Cfl1* mRNA (white) using Stellaris™ RNA FISH. The co-localization of hnRNP Q and *Cfl1* mRNA in soma (1A and 3A) and axon (2A and 4A) of the neuron is shown through scaled-up images. Scale bar = 30 μM. (**H**) Quantification of co-localization between *Cfl1* mRNA and hnRNP Q in normal neuron (white) and cOGD neuron (gray) from experiments in (**G**), measured by the percentage of co-localization (co-localized *Cfl1* mRNA spot/total *Cfl1* mRNA spot) (*n* = 10). (**I**) The co-localization between *Cfl1* mRNA and hnRNP A1 in primary hippocampal neuron that was cultured in normoxia condition (top) and cOGD condition (bottom) was observed by the immunofluorescence labeling of hnRNP A1 (magenta) and Tau (green), along with the RNA FISH of *Cfl1* mRNA (white). The co-localization of hnRNP A1 and *Cfl1* mRNA in soma (1A and 3A) and axon (2A and 4A) of the neuron is shown through scaled-up images. Scale bar = 30 μM. (**J**) Quantification of co-localization between *Cfl1* mRNA and hnRNP A1 in normal neuron (white) and cOGD neuron (gray) from experiments in (I), measured by the percentage of co-localization (co-localized *Cfl1* mRNA spot/total *Cfl1* mRNA spot) (*n* = 8). ** *p* ≤ 0.01, *** *p* ≤ 0.001, **** *p* ≤ 0.0001; unpaired Student’s *t* test was performed for (**B**,**C**,**E**,**F**,**H**,**J**). The “*n*” represents the number of independent experiments. Error bars indicate SDs.

If hnRNP Q and hnRNP A1 do not alter the expression of *Cfl1* in normal primary hippocampal neurons, how about in neurons cultured under cOGD conditions (cOGD neurons)? A previous study showed that the expression and localization of proteins often change under different cellular stresses [26]. Under cOGD condition, the level of cofilin was significantly increased, as expected (Figure 6A,B,D,E). Interestingly, the level of hnRNP Q was significantly decreased (Figure 6A,C), while the level of hnRNP A1 was significantly increased in cOGD neurons (Figure 6D,F).Very surprisingly, the localization of hnRNP Q and hnRNP A1 seemed to change under cOGD conditions.

The changes in the proteins’ localization may have affected the interaction with *Cfl1* mRNA. Thus, we additionally stained *Cfl1* mRNA in a cOGD neuron and observed its co-localization with the proteins. While hnRNP Q was co-localized with *Cfl1* mRNA in both the soma and axon of the control neuron (Figure 6G, box 1A and 2A), it was co-localized in the soma of the cOGD neuron (Figure 6G, box 3A and 4A). Furthermore, the total co-localization between hnRNP Q and *Cfl1* mRNA, as well as its amount in axonal fraction (Figure S6A), was significantly decreased in cOGD neurons (Figure 6H). In regard to hnRNP A1, it interacted with *Cfl1* mRNA only in the nucleus of control neuron (Figure 6I, box 1A and 2A), while it interacted with *Cfl1* mRNA in both the soma and axon of cOGD neurons (Figure 6I, box 3A and 4A). Additionally, the total co-localization between hnRNP A1 and *Cfl1* mRNA, as well as its amount in cytoplasmic fraction (Figure S6B), was significantly increased in the cOGD neuron (Figure 6J). These data demonstrate that, under normal conditions, the strong interaction between hnRNP Q and *Cfl1* mRNA inhibits the interaction between nuclear hnRNP A1 and *Cfl1* mRNA. However, under cOGD conditions, the re-localization of hnRNP Q and hnRNP A1 promotes the interaction between hnRNP A1 and *Cfl1* mRNA, which further increases the translational activity of *Cfl1* mRNA.

### 3.6. Cerebral Ischemia Mouse Model Showed Similar Expression and Localization of hnRNP Q and hnRNP A1

**Figure 7:**
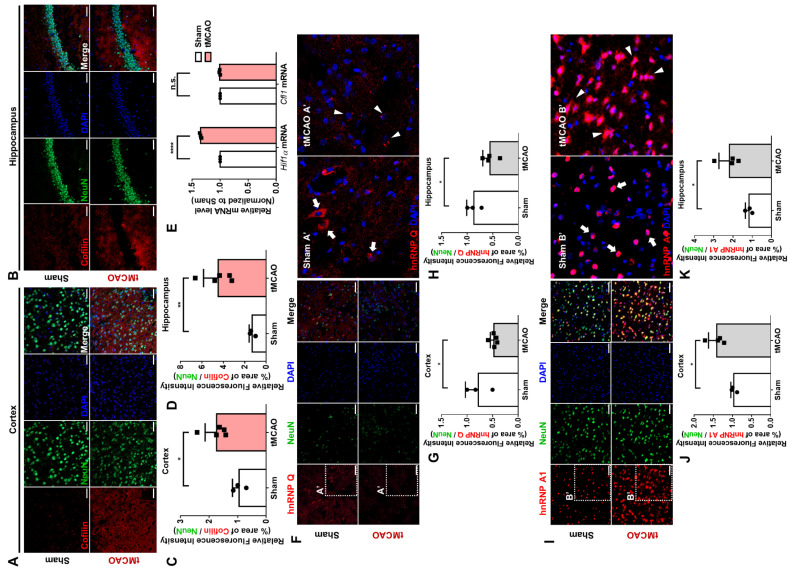
Expression and localization of hnRNP Q and hnRNP A1 in tMCAO mouse. (**A**,**B**) The immunofluorescence labeling of cofilin (red), DAPI (blue), and NeuN (green) in the cortex (**A**) and the hippocampus (**B**) of sham (top) or transiently middle artery occlusion (tMCAO) mouse (bottom). Scale bar = 50 μM. (**C**,**D**) Quantification of relative fluorescence intensity of cofilin in the cortex (**C**) and the hippocampus (**D**) of sham mouse (white) or tMCAO mouse (pink), calculated by the area of intensity, which was normalized to the intensity of NeuN (*n* = 5). (**E**) RT-qPCR analysis of *Hif1α* mRNA or *Cfl1* mRNA level in the brain of sham mouse (white) or tMCAO mouse (pink), normalized to the mRNA level of the sham mouse (*n* = 3). (**F**) The immunofluorescence labeling of hnRNP Q (red), DAPI (blue), and NeuN (green) in the cortex of sham (top) or tMCAO mouse (bottom). The localization of hnRNP Q in the cortex of sham (Sham A’) or tMCAO mouse (tMCAO A’) is shown through scaled-up images. White arrowhead indicates axonal hnRNP Q, while white triangle indicates granule-like hnRNP Q. Scale bar = 50 μM. (**G**,**H**) Quantification of relative fluorescence intensity of hnRNP Q in the cortex (**G**) and the hippocampus (**H**) of sham mouse (white) or tMCAO mouse (gray), calculated by the area of intensity, which was normalized to the intensity of NeuN (*n* = 5). (**I**) The immunofluorescence labeling of hnRNP A1 (red), DAPI (blue), and NeuN (green) in the cortex of sham (top) or tMCAO mouse (bottom). The localization of hnRNP A1 in the cortex of sham (Sham A’) or tMCAO mouse (tMCAO A’) is shown through scaled-up images. White arrowhead indicates nuclear hnRNP A1, while white triangle indicates cytoplasmic hnRNP A1. Scale bar = 50 μM. (**J**,**K**) Quantification of relative fluorescence intensity of hnRNP A1 in the cortex (**J**) and the hippocampus (**K**) of sham mouse (white) or tMCAO mouse (gray), calculated by the area of intensity, which was normalized to the intensity of NeuN (*n* = 5). n.s., not significant, * *p* ≤ 0.05, ** *p* ≤ 0.01, **** *p* ≤ 0.0001; unpaired Student’s *t* test was performed for (**C**,**D**,**G**,**H**,**J**,**K**); two-way ANOVA with Tukey’s multiple comparison test was performed for (**E**). “*n*” represents the number of mice. Error bars indicate SDs.

The cOGD neuron may not truly represent the neurons undergoing degeneration, so we used a cerebral ischemic model mouse, which was induced through transient middle cerebral artery occlusion (tMCAO). It is known that severe neurodegeneration occurs during cerebral ischemia due to transient OGD [5]. When we looked at the level of cofilin, it was significantly increased in the cortex (Figure 7A,C) and the hippocampus (Figure 7B,D) of the tMCAO mouse. On the other hand, while the mRNA level of *Hif1α* was increased in the tMCAO mouse’s brain due to oxidative stress, the mRNA level of *Cfl1* was not altered (Figure 7E). These data indicate that the increase in the level of cofilin is not induced at the transcriptional level but likely at the translational level.

Then, similar to the results from the cOGD neuron, the protein level of hnRNP Q was significantly decreased in the cortex (Figure 7F,G) and the hippocampus (Figure 7H and Figure S7A) of the tMCAO mouse. Intriguingly, we found that hnRNP Q, which was localized in axon and soma of the neuron in the sham mouse cortex (Figure 7F, box Sham A’), was localized as a granule-like spot at the soma of the neuron in the tMCAO mouse cortex (Figure 7F, box tMCAO A’). The level of hnRNP A1 was notably increased in both the cortex (Figure 7I,J) and the hippocampus (Figure 7K and Figure S7B) of the tMCAO mouse. Additionally, while we observed hnRNP A1 mainly in the nucleus of the neuron in the sham mouse cortex (Figure 7I, box Sham B’), we found hnRNP A1 in the cytoplasmic area of the neuron in the tMCAO mouse cortex (Figure 7I, box tMCAO B’). These data, altogether, suggest that the expression and the localization of hnRNP Q and hnRNP A1 in tMCAO mouse are altered in a way that could promote the translation of *Cfl1* mRNA (Figure S7C).

### 3.7. Altering the Level of hnRNP Q and hnRNP A1 in cOGD Neuron Alleviates the Formation of Cofilin–Actin Aggregates

**Figure 8:**
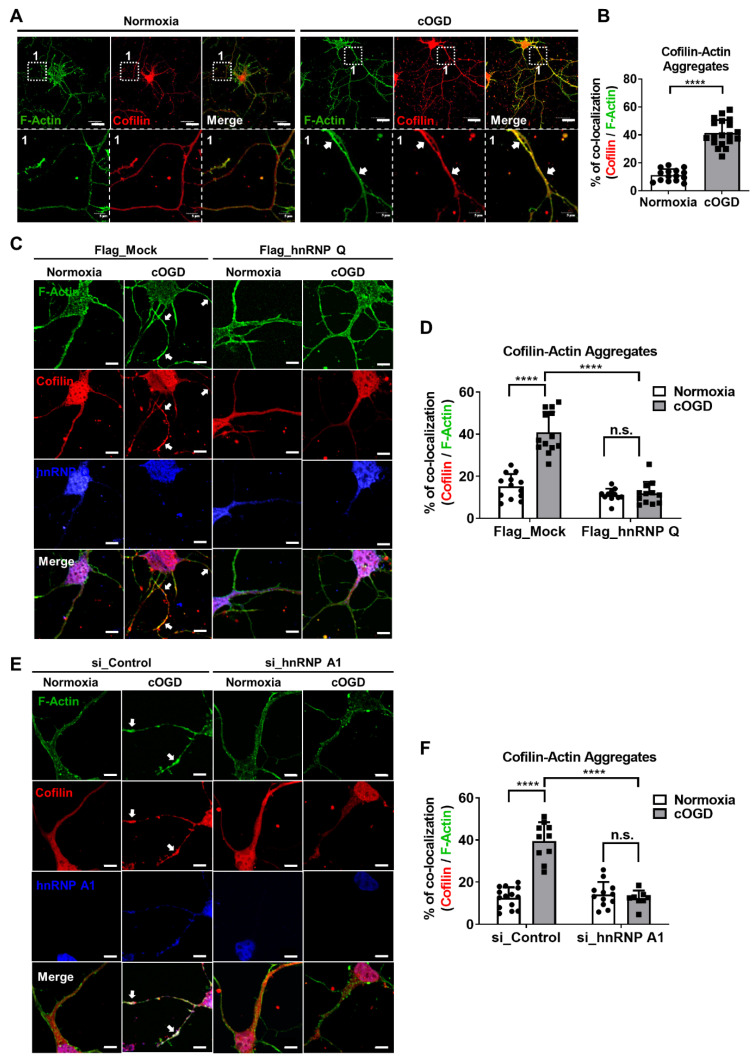
Changing the level of hnRNP Q and hnRNP A1 in cOGD neurons inhibits the formation of cofilin–actin aggregates. (**A**) Cofilin–actin aggregates were stained by immunofluorescence labeling of cofilin (red), and F-actin (green) in the primary hippocampal neuron that was cultured under normoxia conditions (left) and cOGD conditions (right). Cofilin–actin aggregates are indicated by the white arrowheads and are shown in scaled-up images labeled as 1. Scale bar = 30 μM. (**B**) Quantification of cofilin–actin aggregates in primary hippocampal neurons that were cultured under normoxia condition (white) and cOGD conditions (gray) by measuring the co-localization of cofilin and F-actin (*n* = 19). (**C**) Cofilin–actin aggregates were stained as previously in the Flag_Mock-expressed (left) and Flag_hnRNP Q1-expressed (right) primary hippocampal neurons that were cultured in normoxia condition (first and third column) and cOGD condition (second and fourth column). Cofilin–actin aggregates are indicated by the white arrowhead. Scale bar = 5 μM. (**D**) Quantification of cofilin–actin aggregates in Flag_Mock-expressed (left) and Flag_hnRNP Q1-expressed (right) primary hippocampal neuron that was cultured under normoxia conditions (white) and cOGD conditions (gray) (*n* = 13). (**E**) Cofilin–actin aggregates were stained as previously in the si_Control-transfected (left) or si_hnRNP A1-transfected (right) primary hippocampal neuron that was cultured under normoxia conditions (first and third column) and cOGD conditions (second and fourth column). Cofilin–actin aggregates are indicated by the white arrowhead. Scale bar = 5 μM. (**F**) Quantification of cofilin–actin aggregates in si_Control-transfected (left) or si_hnRNP A1-transfected (right) primary hippocampal neuron that was cultured under normoxia conditions (white) and cOGD conditions (gray) (*n* = 14). n.s., not significant, **** *p* ≤ 0.0001; unpaired Student’s *t* test was performed for (**B**); two-way ANOVA with Tukey’s multiple comparison test was performed for (**D**,**F**). The “*n*” represents the number of independent experiments. Error bars indicate SDs.

We have demonstrated that the expression and localization of hnRNP Q and hnRNP A1 are changed under OGD conditions in a way that could promote *Cfl1* mRNA translation. As mentioned previously, the increased expression of *Cfl1* can induce cofilin–actin aggregate formation [12]. Therefore, we wondered whether changing the expression of hnRNP Q or hnRNP A1 could alleviate the aggregate formation. We co-stained cofilin and filamentous actin (F-actin) and observed whether cofilin co-localizes with F-actin as aggregates. We found that the aggregate formation was significantly induced in cOGD neurons (Figure 8A,B). Then, we altered the level of hnRNP Q and hnRNP A1 in the cOGD neurons to inhibit the *Cfl1* mRNA translation. As expected, the cofilin–actin aggregate formation in the cOGD neuron was significantly alleviated by the overexpression hnRNP Q (Figure 8C,D and Figure S7D) and by the knockdown of hnRNP A1 (Figure 8E,F and Figure S7E). Altogether, we demonstrated that the altered expression and localization of hnRNP Q and hnRNP A1 during OGD condition promotes the translation of *Cfl1* mRNA and thus the formation of aggregates.

## 4. Discussion

Although a relationship between the expression of *Cfl1* and the cofilin–actin aggregates formation was previously suggested [12], the regulatory mechanism behind the expression of *Cfl1* under OGD conditions was unclear. Here, we revealed an important regulatory mechanism of *Cfl1* expression during OGD that could promote the formation of the cofilin aggregates. We initially found that the translational activity of *Cfl1* mRNA is increased in cOGD neurons and that two RNA-binding proteins, hnRNP Q and hnRNP A1, interact with the 5′UTR of *Cfl1* mRNA at similar locations. However, the mechanism behind how these proteins bind at similar locations of *Cfl1* mRNA is yet to be found. Previous studies revealed that RNA-binding proteins usually interact with specific motif sequences of RNA [27]. In this case, hnRNP Q and hnRNP A1 may share similar binding motifs, which exist on the D1 region of *Cfl1* mRNA. Additionally, this may be why less hnRNP A1 interact with *Cfl1* mRNA in the presence of hnRNP Q. The binding motif for hnRNP A1 may not be available when *Cfl1* mRNA is bound by hnRNP Q, a relatively bigger-sized protein. Two proteins may also form a complex, like other RNA-binding protein complexes that regulate the translation [16,17]. In the presence of hnRNP Q, hnRNPA1 may be recruited to hnRNP Q, instead of *Cfl1* 5′UTR. All in all, further investigations are needed to reveal how they interact at *Cfl1* mRNA.

We further illustrated that hnRNP Q inhibits the interaction between hnRNP A1 and *Cfl1* mRNA and further suppresses the translational activity of *Cfl1* mRNA. However, there was a clear discrepancy in this mechanism between n2a cells and primary hippocampal neurons. Unlike the results found with n2a cells, hnRNP Q or hnRNP A1 did not alter the level of cofilin nor the translation of *Cfl1* 5′UTR in primary hippocampal neurons. This divergence may come from differences in the characteristics of the cell line and primary cells. Many times, the scientists use the immortalized cells because they are easier to maintain and use during experiments. However, the problem with the cells is that they are sometimes misidentified over multiple passage of culturing [22,23]. Additionally, the environment in which the cells are cultured is often different from the actual tissue which they were isolated from. This difference may affect the whole protein profiling of the cells and reduce its biological relevance [24,25]. This may be the case with hnRNP A1 in n2a cell and primary hippocampal neurons. In n2a cells, hnRNP A1 localizes in both the nucleus and cytoplasm, while it mainly localizes in the nucleus of neurons. Due to the difference, the interaction between hnRNP A1 and *Cfl1* mRNA could not have been increased by the knockdown of hnRNP Q in neurons since there is almost no hnRNP A1 to interact with in the cytoplasm.

We then demonstrated that hnRNP Q and hnRNP A1 re-localizes under stress caused by OGD and alters the co-localization between the proteins and *Cfl1* mRNA. The interaction between hnRNP A1 and *Cfl1* mRNA especially increases significantly under cOGD conditions. We found that hnRNP Q re-localizes to the granule-like spots in the soma, while hnRNP A1 re-localizes from the nucleus to the soma and axon. Previous studies show that proteins may change their localization under high oxidative stress conditions [28,29]. A study revealed that arsenite-induced oxidative stress could induce the cytoplasmic re-localization of hnRNP A1, which then participates in mRNA translation [28]. Others revealed that oxidative stress re-localizes hnRNP Q from the axonal transport granule to the cytoplasmic stress granule located in the soma [29]. Since cOGD neurons and the tMCAO mouse mimic the stressful environment of neurodegeneration, hnRNP Q and hnRNP A1 could surely change their location. Additionally, we cannot exclude the effects of glial cells on the response of hippocampal neurons to a stressful environment. Although we added cytosine arabinoside (AraC) to minimize the effect of glial cells, we could not completely eliminate them. Thus, further experiments examining the mechanism behind the re-localization of proteins, which may be responses of neurons to a stressful environment, should be conducted.

Finally, we showed that altering the expression of hnRNP Q or hnRNP A1 to inhibit the translation of *Cfl1* mRNA significantly alleviates the formation of cofilin–actin aggregates. Cofilin–actin aggregates are toxic to neurons as they can induce neurodegeneration by interrupting the intracellular vesicular transport system and synaptic function [12,30]. These toxic aggregates are transiently formed under OGD conditions, such as an ischemic injury [31]. Our study revealed a key mechanism behind the formation of the toxic aggregations, which suggests a possible therapeutic approach. We found that increasing the level of hnRNP Q or decreasing the level of hnRNP A1 inhibits the formation of cofilin–actin aggregates. Like other RNAi systems used in different disorders [32,33], RNAi therapy targeting hnRNP A1 may alleviate the aggregate formation in the neurons and prevent the neurodegeneration. Altogether, our study revealed a novel regulatory mechanism behind the translation of *Cfl1* mRNA and the formation of cofilin–actin aggregates during oxygen–glucose deprivation.

## Data Availability

All data are available in the main text or the Supplementary Materials.

## Supplementary Materials

The following are available online at https://www.mdpi.com/article/10.3390/cells10123567/s1, Figure S1: The translational activity of *Cfl1* mRNA is enhanced in cOGD neuron, Figure S2: HnRNP Q1 and hnRNP A1 regulates the translational activity of *Cfl1* mRNA, Figure S3: HnRNP Q1 interrupts the interaction between hnRNP A1 and *Cfl1* mRNA, Figure S4: Interaction between hnRNP Q1 and hnRNP A1 is independent from Nptb, Figure S5: The protein level of Cofilin in primary hippocampal neuron is unaffected by hnRNP Q1 and hnRNP A1 under normal condition, Figure S6: Re-localization of hnRNP Q and hnRNP A1 in hippocampal neuron, Figure S7: Expression of hnRNP Q and hnRNP A1 in tMCAO hippocampus and plasmid transfected cOGD neuron.
