# Supplementary material for: hnRNP Q and hnRNP A1 Regulate the Translation of Cofilin in Response to Transient Oxygen–Glucose Deprivation in Hippocampal Neurons
Source: Cells. 2021 Dec 17;10(12):3567. doi: 10.3390/cells10123567 (PMC8700186; doi:10.3390/cells10123567)
Supplement: Supplementary file 1 [file cells-10-03567-s001.zip › cells-1499230-supplementary.pdf]

Supplementary Material for

“hnRNP Q and hnRNP A1 regulate the formation of cofilin-actin rod during cerebral ischemia”

Sung Wook Kim, In Kyung Hong, Mingee Kim, Yun Seon Song*, Kyong-Tai Kim*

*Corresponding authors. Email: yssong@sookmyung.ac.kr and ktk@postech.ac.kr

This supplementary material includes

Supplementary Figure Legends

Figure S1~S6

Supplementary Figure Legends

Figure. S1. The translational activity of *Cfl1* mRNA is enhanced in cOGD neuron. (**A**) Illustration of bicistronic vector that we used to measure the translational activity of *Cfl1* mRNA 5'UTR in primary hippocampal neuron. 5'UTR sequences of *Cfl1* mRNA (gray) was inserted in between the sequences of mCherry (red) and eGFP (green). (**B**) Measuring the translational activity of *Cfl1* mRNA using mCherry (red) – eGFP (green) bicistronic vector with no 5'UTR (left), *Cfl1* 5'UTR (middle) or reverse form of *Cfl1* 5'UTR (right), in primary hippocampal neuron. The fluorescent signal of eGFP was only observable in bicistronic vector with *Cfl1* 5'UTR. Scale bar = 10μM.

Figure. S2. HnRNP Q1 and hnRNP A1 regulates the translational activity of *Cfl1* mRNA. (**A**) Table of proteins that are bound to 5'UTR of *Cfl1* mRNA found through Orbitrap (n=1). (**B**) Representative Western blot of cofilin during hnRNP Q1 or hnRNP Q total knockdown experiment. N2a cells were transfected with si-control (first lane), si-hnRNP Q1 (second lane) or si-hnRNP Q total (third lane). Each isoforms of hnRNP Q and hnRNP R are indicated by black arrowheads. GAPDH was used as loading control. (**C**) Representative Western blot of hnRNP Q in primary hippocampal neuron (1: 30μg of lysate; 2: 15μg of lysate). Each isoforms of hnRNP Q and hnRNP R are indicated by black arrowheads. GAPDH was used as loading control. (**D**) Quantification of protein level of hnRNP Q1 (left), hnRNP A1 (middle) and cofilin (right) in n2a cell lines transfected with si-control (gray), si-hnRNP A1 (orange), si-hnRNP Q1 (blue), or si-hnRNP A1 / si-hnRNP Q1 (green) from Figure 2E. ImageJ was used to measure the intensity of the blot. n.s., not significant, $^{**}P \leq 0.01$, $^{***}P \leq 0.001$, and $^{****}P \leq 0.0001$; two-way ANOVA with Tukey's multiple comparison test (n=3). (E) Relative mRNA level of *Cfl1* (left), *Hnrnpa1* (middle), or *Syncrip* (right) in n2a cell lines transfected with si-control (gray), si-hnRNP A1 (orange), si-hnRNP

Q1 (blue), or si-hnRNP A1 / si-hnRNP Q1 (green) from Figure 2I, measured by RT-qPCR (n=3). Data are represented as ± SD.

Figure. S3. HnRNP Q1 interrupts the interaction between hnRNP A1 and *Cfl1* mRNA. (**A**) Representative immunoblot image of immunoprecipitation during RNA immunoprecipitation assay using normal anti-mouse IgG (middle) or anti-hnRNP Q (right). Heavy chain or light chain were used to normalize the intensity hnRNP Q1. (**B**) Representative immunoblot image of immunoprecipitation during RNA immunoprecipitation assay using normal anti-mouse IgG (middle) or anti-hnRNP A1 (right). Heavy chain was used to normalize the intensity hnRNP A1. (**C**) The secondary structure of 5'UTR of Cfl1 mRNA (145 nucleotides) were generated using mfold software. D1 region, where IRES element of Cfl1 mRNA exist, is highlighted in red, while D2 region is highlighted in green. (**D**) Representative blot image of *in vitro* RNA binding assay that was performed to measure the binding pattern of hnRNP A1 during the knockdown of hnRNP Q1 or hnRNP Q total. *In vitro* transcribed *Cfl1* 5'UTR was biotinylated before the incubation in the lysates of n2a cells that were transfected with si-control (left three lanes), si-hnRNP Q1 (middle three lanes), or si-hnRNP Q total (right three lanes) and pulled down using streptavidin. Each isoforms of hnRNP Q and hnRNP R are indicated by black arrowheads. GAPDH was used as loading control. (**E**) Representative blot image of *in vitro* RNA binding assay that was performed to measure the binding pattern of hnRNP Q1 during the knockdown of hnRNP A1. *In vitro* transcribed *Cfl1* 5'UTR was incubated in the lysates of n2a cell line that were transfected with si-control (left three lanes) or si-hnRNP A1 (right three lanes), and pulled down using streptavidin. 14-3-3ζ was used as loading control. (**F**) Quantification of the interaction between hnRNP Q1 and *Cfl1* 5'UTR in n2a cell lines transfected with si-control (gray) or si_hnRNP A1 (orange) from experiment performed in (E). The interaction between hnRNP

A1 and *Cfl1* 5'UTR was normalized to the hnRNP A1 of the input (first lane), which was normalized by 14-3-3ζ. ImageJ was used to measure the intensity of the blot. n.s., not significant; unpaired Student's t test (n=3). Data are represented as ± SD. (**G**) Representative immunoblot image of immunoprecipitation during RNA immunoprecipitation assay using normal anti-mouse IgG or anti-hnRNP A1 in n2a cells (left three lanes) or hnRNP Q1 KO cells (right three lanes). Heavy chain was used to normalize the intensity hnRNP A1.

Figure. S4. Interaction between hnRNP Q1 and hnRNP A1 is independent from nPTB. (**A**) Representative blot image of *in vitro* RNA binding assay that was performed to measure the binding pattern of nPTB in hnRNP Q1 KO cell line. *In vitro* transcribed *Cfl1* 5'UTR was biotinylated before incubation in the lysates of n2a cell line (left three lanes) or hnRNP Q1 KO cell line (right three lanes), and was pulled down using streptavidin. The immunoblot of nPTB is boxed in pink. (**B**) Quantification of the interaction between nPTB and *Cfl1* 5'UTR in n2a cell lines (gray) or hnRNP Q1 KO cell line (pink) from experiment performed in (A). The interaction between nPTB and *Cfl1* 5'UTR was normalized to the nPTB of the input (first lane) which was normalized by 14-3-3ζ. ImageJ was used to measure the intensity of the blot. n.s., not significant; unpaired Student's t test (n=3). Data are represented as ± SD. (**C**) Representative Western blot of nPTB (boxed in pink) during nPTB knockdown experiment. N2a cells were transfected with si-control (first lane) or si-nPTB (second lane). 14-3-3ζ was used as the loading control. (**D**) Representative blot image of *in vitro* RNA binding assay that was performed to measure the binding pattern of hnRNP Q1 and hnRNP A1 during nPTB knockdown. *In vitro* transcribed *Cfl1* 5'UTR was biotinylated before incubation in the lysates of n2a cell line transfected with si-control (left three lanes) or si-nPTB (right three lanes), and was pulled down using streptavidin. The immunoblot of nPTB

is boxed in pink and the black arrowhead indicates hnRNP Q1. 14-3-3ζ was used as the loading control.

Figure. S5. The protein level of Cofilin in primary hippocampal neuron is unaffected by hnRNP Q1 and hnRNP A1 under normal condition. (**A**) Change in the protein level of cofilin during the knockdown of hnRNP Q1 was observed by the immunofluorescence labelling of cofilin (blue), hnRNP Q (red), and GFP (green) in primary hippocampal neuron that was transfected with sh_Mock (left) or sh_hnRNP Q1 (right). White triangle or arrow head indicates the neuron with shRNA expression, while yellow triangle or arrow head indicates the neuron without shRNA expression. Scale bar = 20μM. (**B**) Quantification of relative fluorescence intensity of cofilin (left) and hnRNP Q (right), calculated by the total fluorescent area per cell, in GFP- hippocampal neuron (gray) or GFP+ hippocampal neuron (green or blue) that were transfected with sh_Mock. n.s., not significant; unpaired Student's t test (n=14). (**C**) Quantification of relative fluorescence intensity of cofilin (left) and hnRNP Q (right), calculated by the total fluorescent area per cell, in GFP- hippocampal neuron (gray) or GFP+ hippocampal neuron (green or blue) that were transfected with sh_hnRNP Q. n.s., not significant; unpaired Student's t test (n=12). (**D**) Change in the protein level of cofilin during the knockdown of hnRNP A1 was observed by the immunofluorescence labelling of cofilin (blue), hnRNP A1 (red), and GFP (green) in primary hippocampal neuron that was transfected with sh_Mock (left) or sh_hnRNP A1 (right). White triangle or arrow head indicates the neuron with shRNA expression, while yellow triangle or arrow head indicates the neuron without shRNA expression. Scale bar = 20μM. (**E**) Quantification of relative fluorescence intensity of cofilin (left) and hnRNP A1 (right), calculated by the total fluorescent area per cell, in GFP- hippocampal neuron (gray) or GFP+ hippocampal neuron (green or orange) that were transfected with sh_Mock. n.s., not significant; unpaired

Student's t test (n=12). (**F**) Quantification of relative fluorescence intensity of cofilin (left) and hnRNP A1 (right), calculated by the total fluorescent area per cell, in GFP- hippocampal neuron (gray) or GFP+ hippocampal neuron (green or orange) that were transfected with sh_hnRNP A1. n.s., not significant; unpaired Student's t test (n=11). Data are represented as ± SD.

Figure. S6. Expression of hnRNP Q and hnRNP A1 in tMCAO hippocampus and plasmid transfected cOGD neuron (**A**) The immunofluorescence labelling of hnRNP Q (red), DAPI (blue) and NeuN (green) in the hippocampus of sham (top) or tMCAO mouse (bottom). Scale bar = 50μM. (**B**) The immunofluorescence labelling of hnRNP A1 (red), DAPI (blue) and NeuN (green) in the hippocampus of sham (top) or transiently middle artery occlusion (tMCAO) mouse (bottom). Scale bar = 50μM. **(C**) Measurement of hnRNP Q in Flag_Mock expressed (left) or Flag_hnRNP Q1 expressed (right) primary hippocampal neuron that was cultured in normoxia condition (white) or cOGD condition (gray). The expression of F-Actin was used for normalization. ***$P \leq 0.001$, ****$P \leq 0.0001$; n.s., not significant; two-way ANOVA with Tukey's multiple comparison test (n=13). (**D**) Measurement of hnRNP A1 in si_Control transfected (left) or si_hnRNP A1 transfected (right) primary hippocampal neuron that was cultured in normoxia condition (white) or cOGD condition (gray). The expression of F-Actin was used for normalization. ****$P \leq 0.0001$; n.s., not significant; two-way ANOVA with Tukey's multiple comparison test (n=14). Data are represented as ± SD.

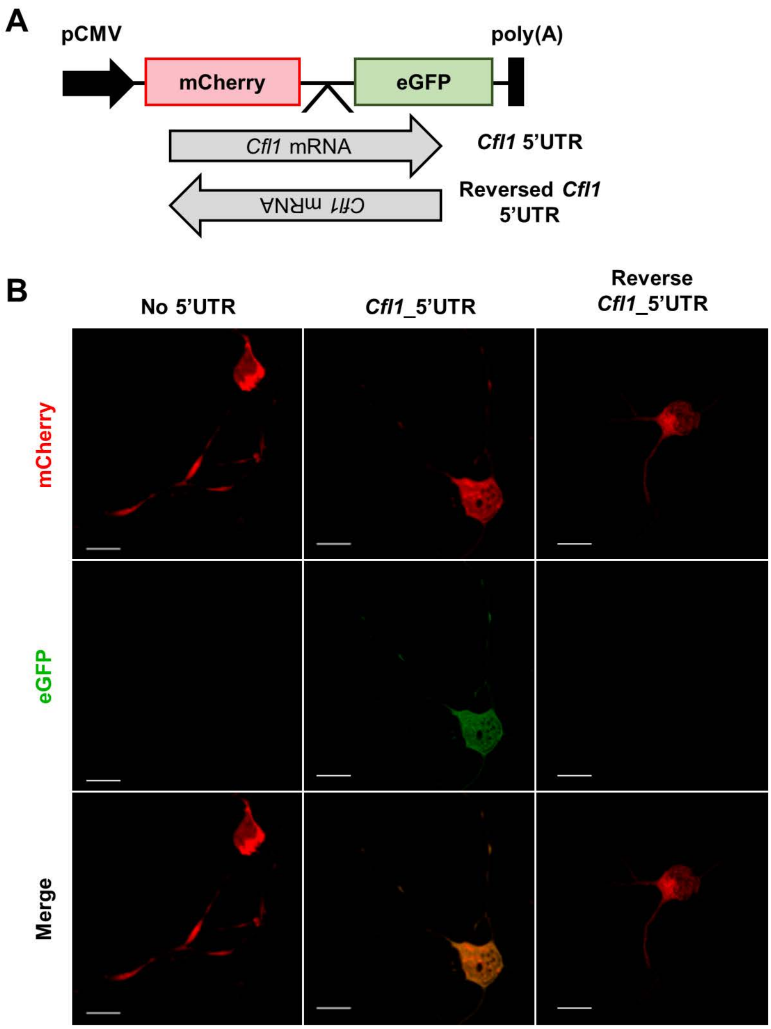

Supplementary Figure S1

A

Orbitrap with *Cfl1* 5'UTR

Accession	MW [kDa]	Calc. pI	Score	Description
P62960	35.7	9.88	33.50	Ybox 1
P49312	34.2	9.23	13.23	HnRNP A1
G3V018	65.7	8.60	2.22	HnRNP Q
G5E8R3	129.6	6.71	33.19	Pyruvate carboxylase
Q9JJZ2	50.0	5.10	17.12	Tubulin alpha-8
Q91Z31	57.5	8.66	7.82	PTBP2

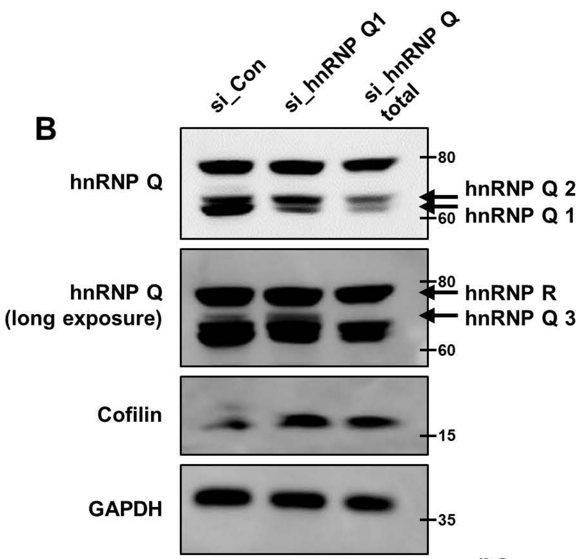

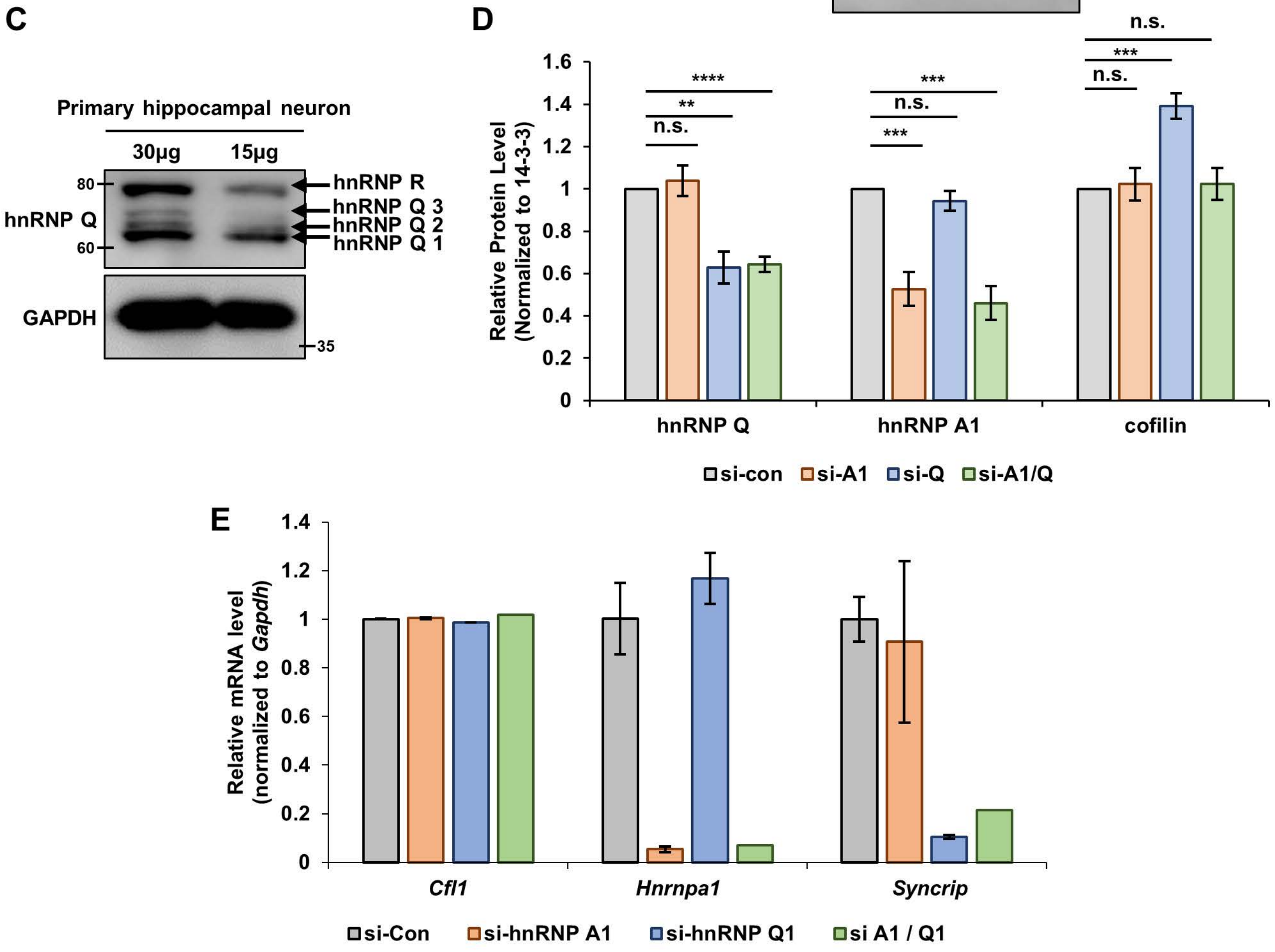

Supplementary Figure S2

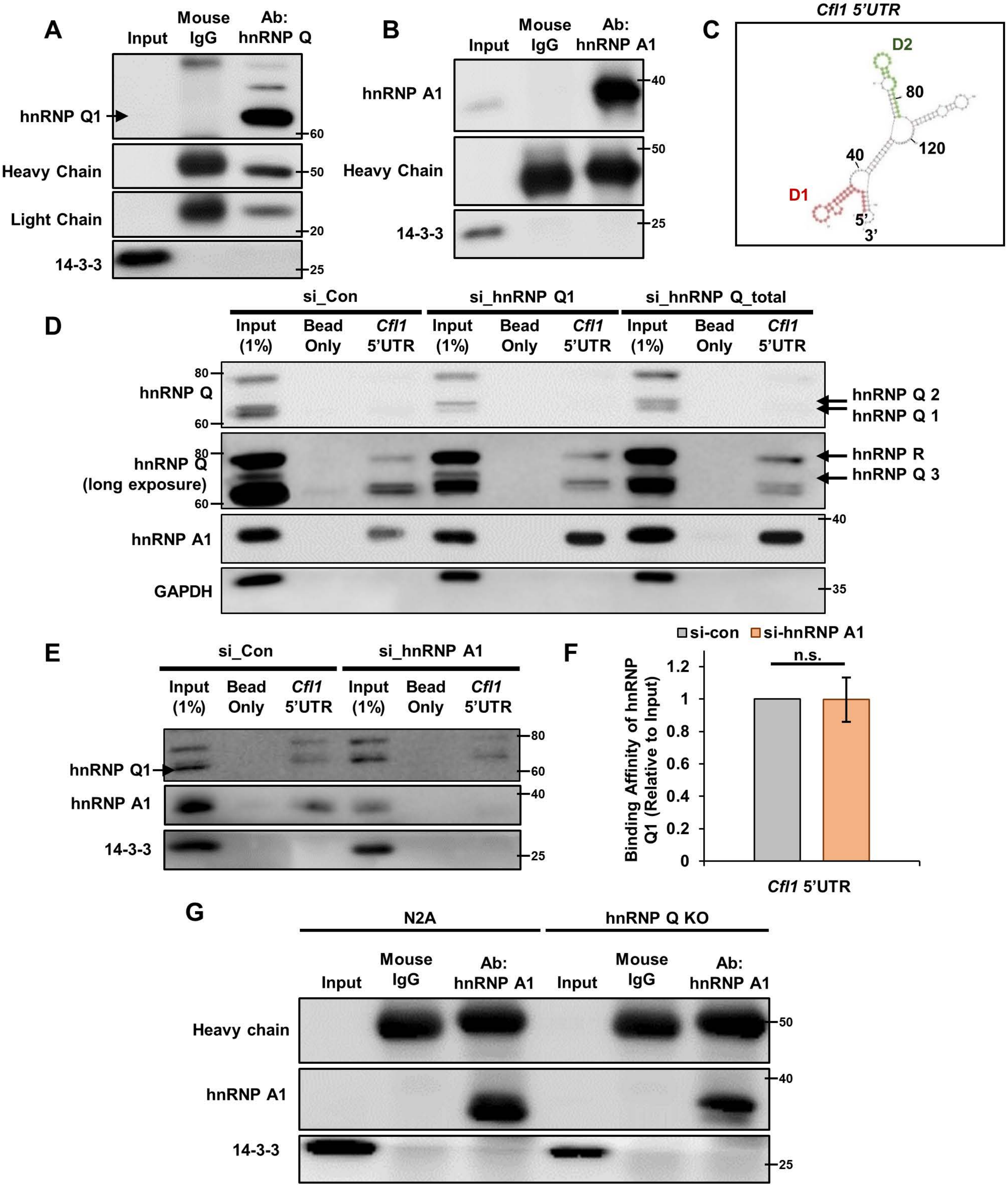

Supplementary Figure S3

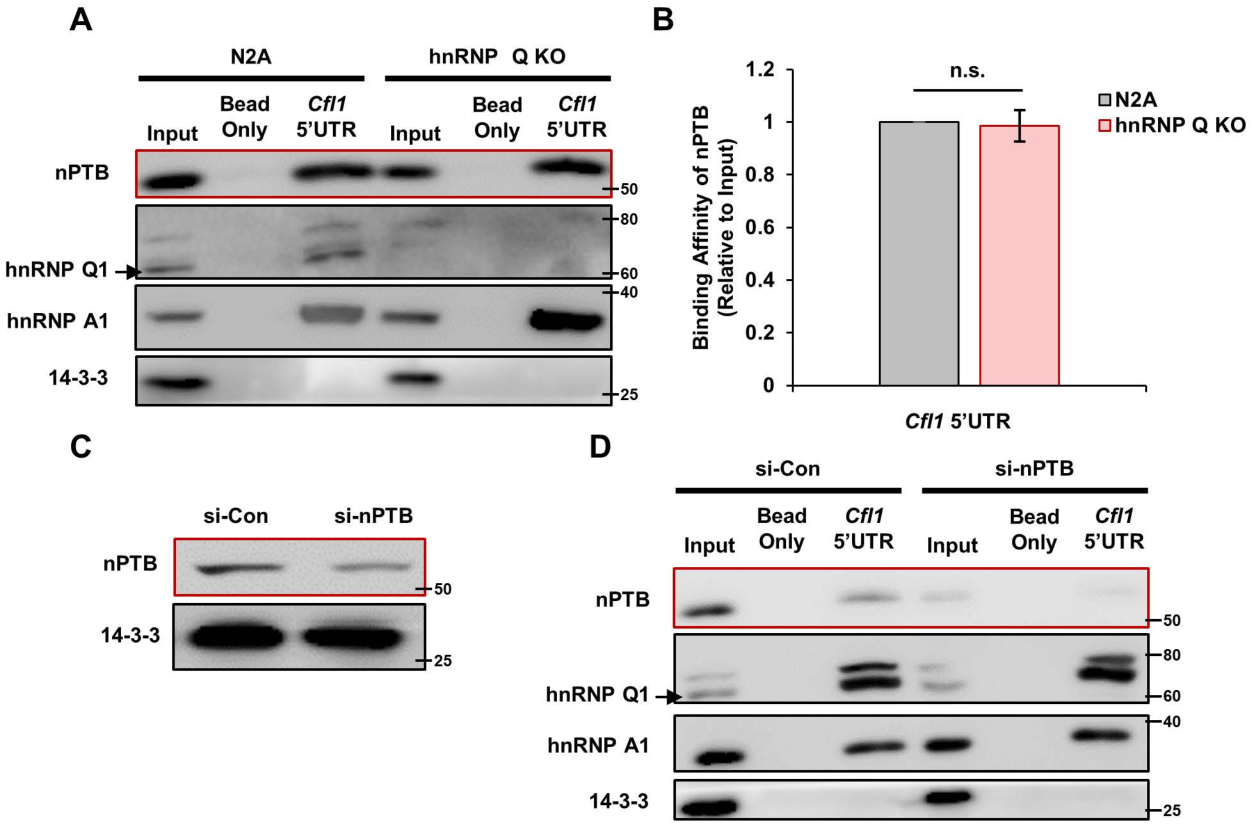

Supplementary Figure S4

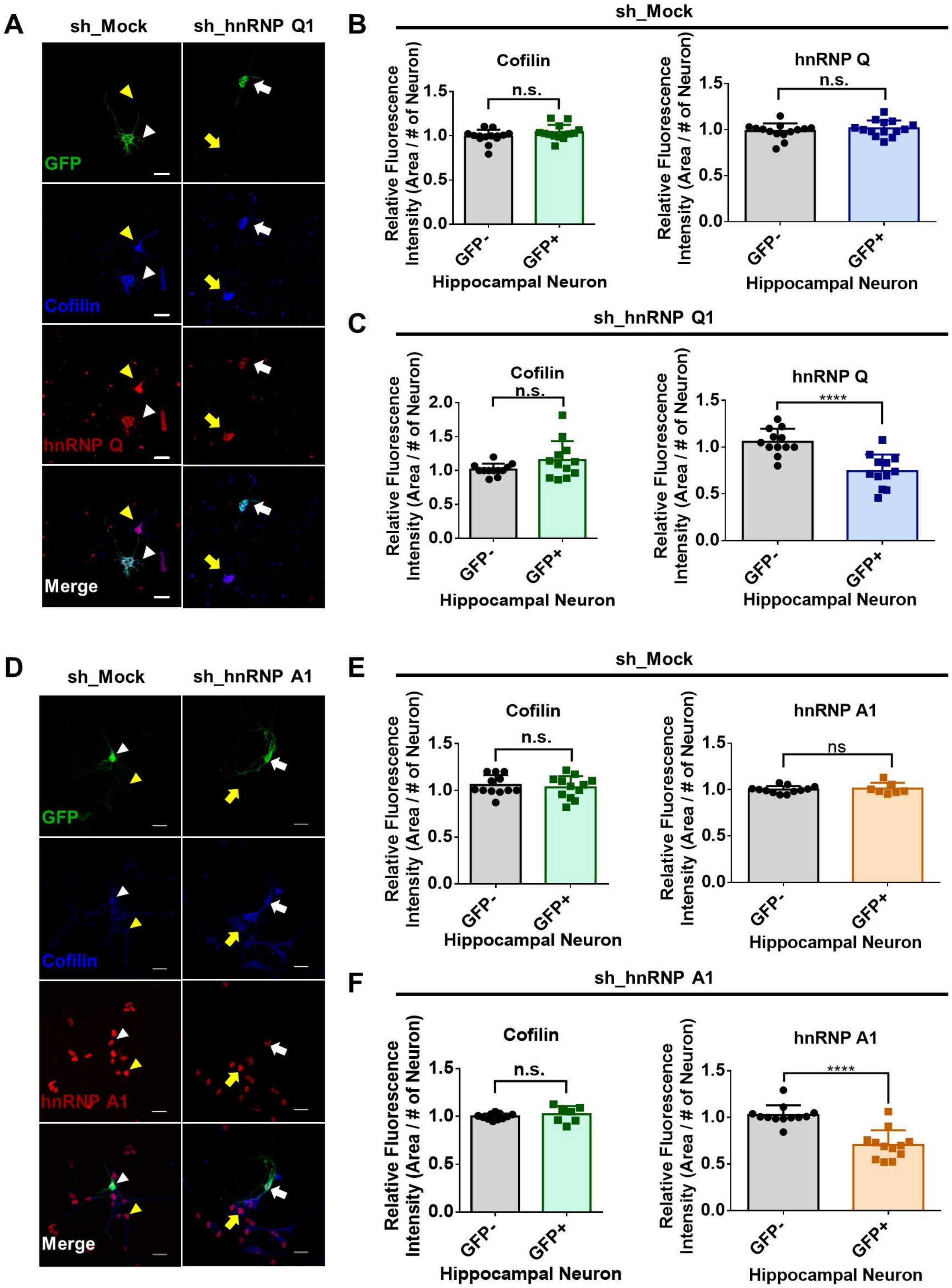

Supplementary Figure S5

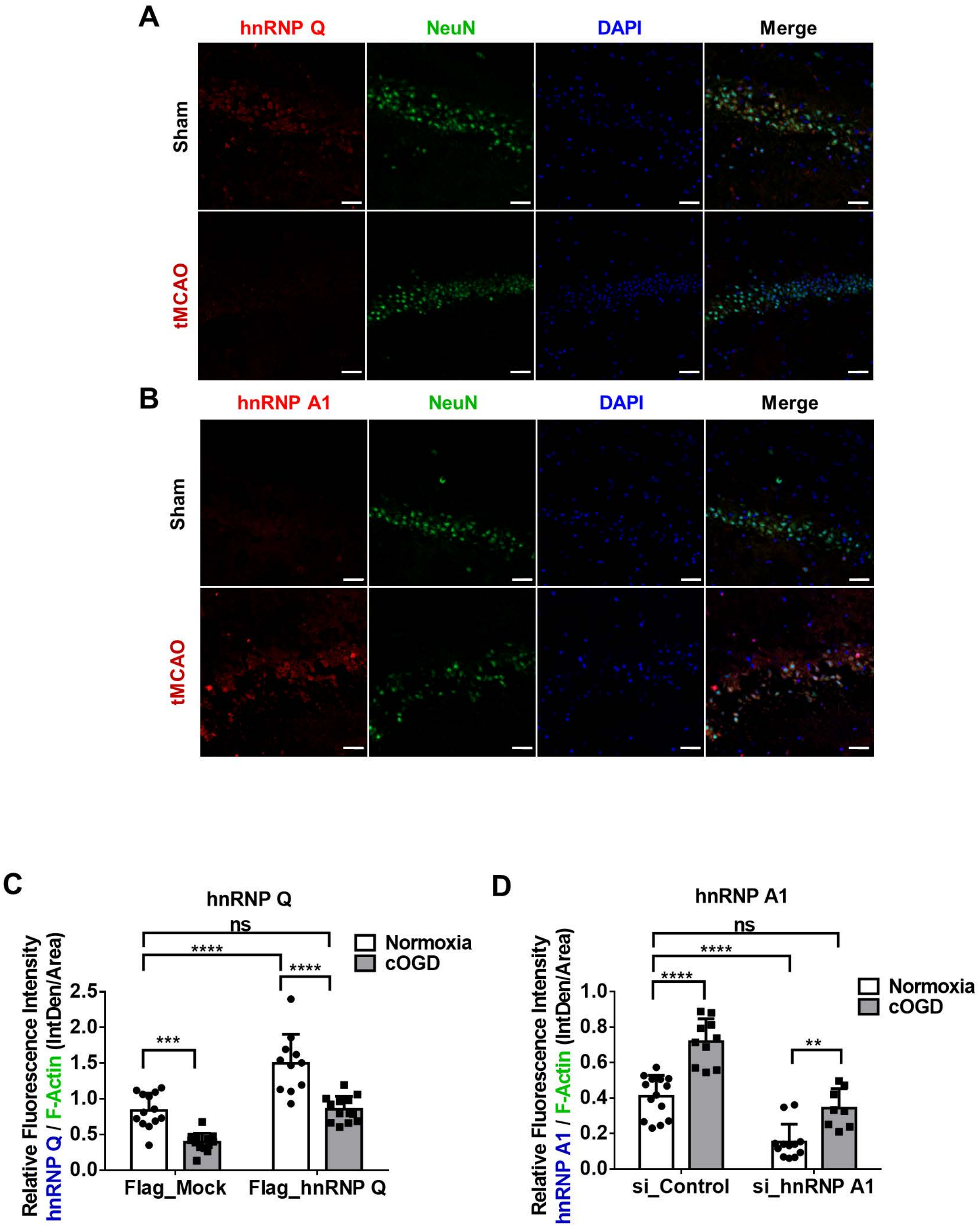

Supplementary Figure S6
